# RAD51AP1 and RAD54L Can Underpin Two Distinct RAD51-Dependent Routes of DNA Damage Repair *via* Homologous Recombination

**DOI:** 10.3389/fcell.2022.866601

**Published:** 2022-05-16

**Authors:** Platon Selemenakis, Neelam Sharma, Mollie E. Uhrig, Jeffrey Katz, Youngho Kwon, Patrick Sung, Claudia Wiese

**Affiliations:** ^1^ Department of Environmental and Radiological Health Sciences, Colorado State University, Fort Collins, CO, United States; ^2^ Cell and Molecular Biology Graduate Program, Colorado State University, Fort Collins, CO, United States; ^3^ Department of Biochemistry and Structural Biology, University of Texas Health Science Center at San Antonio, San Antonio, TX, United States

**Keywords:** homologous recombination, replication, genome stability, genetic interaction, RAD51AP1, RAD54L, RAD54B

## Abstract

Homologous recombination DNA repair (HR) is a complex DNA damage repair pathway and an attractive target of inhibition in anti-cancer therapy. To help guide the development of efficient HR inhibitors, it is critical to identify compensatory HR sub-pathways. In this study, we describe a novel synthetic interaction between RAD51AP1 and RAD54L, two structurally unrelated proteins that function downstream of the RAD51 recombinase in HR. We show that concomitant deletion of *RAD51AP1* and *RAD54L* further sensitizes human cancer cell lines to treatment with olaparib, a Poly (adenosine 5′-diphosphate-ribose) polymerase inhibitor, to the DNA inter-strand crosslinking agent mitomycin C, and to hydroxyurea, which induces DNA replication stress. We also show that the RAD54L paralog RAD54B compensates for RAD54L deficiency, although, surprisingly, less extensively than RAD51AP1. These results, for the first time, delineate RAD51AP1- and RAD54L-dependent sub-pathways and will guide the development of inhibitors that target HR stimulators of strand invasion.

## Introduction

Homologous recombination (HR) is an essential DNA damage repair pathway critical for genome stability and tumor suppression. HR is altered in many different tumor types and has become an attractive target for the development of new anti-cancer therapies (Li et al., 2017; Kopa et al., 2019; Trenner and Sartori, 2019). Accurate HR is restricted to S- and G2- phases of the cell cycle, and the sister chromatid is used as the template for the restoration of lost sequence information at the damaged DNA site. At the DNA break, a 3′-single-stranded (ss)DNA overhang is generated and protected by the ssDNA-binding protein RPA (Symington, 2014; Daley et al., 2015). RPA is replaced by the RAD51 recombinase, a rate-limiting step in the HR reaction that is facilitated by multiple recombination mediators (Sung, 1997a; Sung, 1997b; Dosanjh et al., 1998; Sung et al., 2003; Zhao et al., 2015; Belan et al., 2021; Roy et al., 2021). The RAD51-ssDNA nucleoprotein filament then catalyzes the capture of the DNA template and initiates the formation of a displacement loop (D-loop) with the assistance of several RAD51-associated proteins (Petukhova et al., 1998; Tanaka et al., 2000; Miyagawa et al., 2002; Modesti et al., 2007; Wiese et al., 2007; Zhao et al., 2017).

RAD51AP1 and RAD54L are two RAD51-associated proteins that co-operate with the RAD51 filament in the capture of the DNA donor molecule and in formation of the D-loop (Petukhova et al., 1998; Tanaka et al., 2000; Miyagawa et al., 2002; Modesti et al., 2007; Wiese et al., 2007; Zhao et al., 2017). RAD51AP1 may have evolved in response to the higher complexities of vertebrate genomes (Parplys et al., 2014). In contrast, RAD54L is highly conserved across eukaryotes (Clever et al., 1997; Essers et al., 1997; Golub et al., 1997; Petukhova et al., 1998; Swagemakers et al., 1998). RAD51AP1 functions in the protection of cells from genotoxic agents, in maintaining genome stability, in the HR-mediated alternative lengthening of telomeres (ALT) pathway and promotes HR when local transcription is active (Henson et al., 2006; Modesti et al., 2007; Wiese et al., 2007; Barroso-Gonzalez et al., 2019; Ouyang et al., 2021). Similarly, RAD54L maintains HR capability, cell survival after treatment with chemotherapeutic agents, and ALT activity (Swagemakers et al., 1998; Tan et al., 1999; Mason et al., 2015; Spies et al., 2016; Mason-Osann et al., 2020). Strikingly, in human cells, loss of either RAD51AP1 or RAD54L engenders only mild HR deficiency (Henson et al., 2006; Modesti et al., 2007; Wiese et al., 2007; Gottipati et al., 2010; Spies et al., 2016; Olivieri et al., 2020).

The RAD51AP1 and RAD54L proteins are unrelated structurally, but both upregulate RAD51 activity by enhancing the ability of the RAD51 filament to engage with the homologous double-stranded (ds)DNA donor (i.e., in synapsis) and in strand invasion (Petukhova et al., 1998; Petukhova et al., 1999; Solinger et al., 2001; Solinger and Heyer, 2001; Sigurdsson et al., 2002; Modesti et al., 2007; Wiese et al., 2007). In these steps of the HR reaction, RAD51AP1 may serve as an anchor between the two DNA molecules undergoing exchange (Modesti et al., 2007; Dunlop et al., 2012; Pires et al., 2021). In contrast, RAD54L belongs to the SWI2/SNF2 protein family of DNA-dependent ATPases (Flaus et al., 2006) and utilizes its ATPase activity to convert the synaptic complex into a D-loop (Sigurdsson et al., 2002; Crickard et al., 2020), and to translocate along the DNA (Van Komen et al., 2000; Ristic et al., 2001) whereby chromatin is remodeled and the turnover of RAD51 is facilitated (Alexiadis and Kadonaga, 2002; Alexeev et al., 2003; Jaskelioff et al., 2003; Li and Heyer, 2009).

The mild phenotype of RAD54L-deficient human cells has been attributed to the existence of RAD54B, a RAD54L paralog (Hiramoto et al., 1999). Human RAD54L and RAD54B share 48% identity and 63% similarity (Flaus et al., 2006; Ceballos and Heyer, 2011). Although less well understood than RAD54L, existing evidence implicates RAD54B in the core mechanisms of HR (Tanaka et al., 2000; Miyagawa et al., 2002; Flaus et al., 2006; McManus et al., 2009; Ceballos and Heyer, 2011). Compared to RAD54L, RAD54B was identified as the weaker ATPase, and these results suggest that RAD54B may fulfil a backup role for RAD54L (Tanaka et al., 2002).

In this study, we show that loss of *RAD54L* in human cells is compensated for by the RAD51AP1 protein. We show that simultaneous deletion of the *RAD54L* and *RAD51AP1* genes further sensitizes human cancer cell lines to treatment with the DNA inter-strand crosslinking agent mitomycin C (MMC), to prolonged exposure to replication stalling by hydroxyurea (HU), and to Poly (adenosine 5′-diphosphate-ribose) polymerase inhibition (PARPi). We also show that the RAD54L paralog RAD54B can substitute for RAD54L activity, but, surprisingly, to a lesser degree than RAD51AP1. Based on these results, we conclude that the activities of RAD51AP1 and RAD54L can underpin two major, mechanistically distinct routes for the completion of HR in human cells.

## Results

### Deletion of Both *RAD54L* and *RAD51AP1* Further Sensitizes Human Cancer Cell Lines to MMC and Olaparib

**FIGURE 1:**
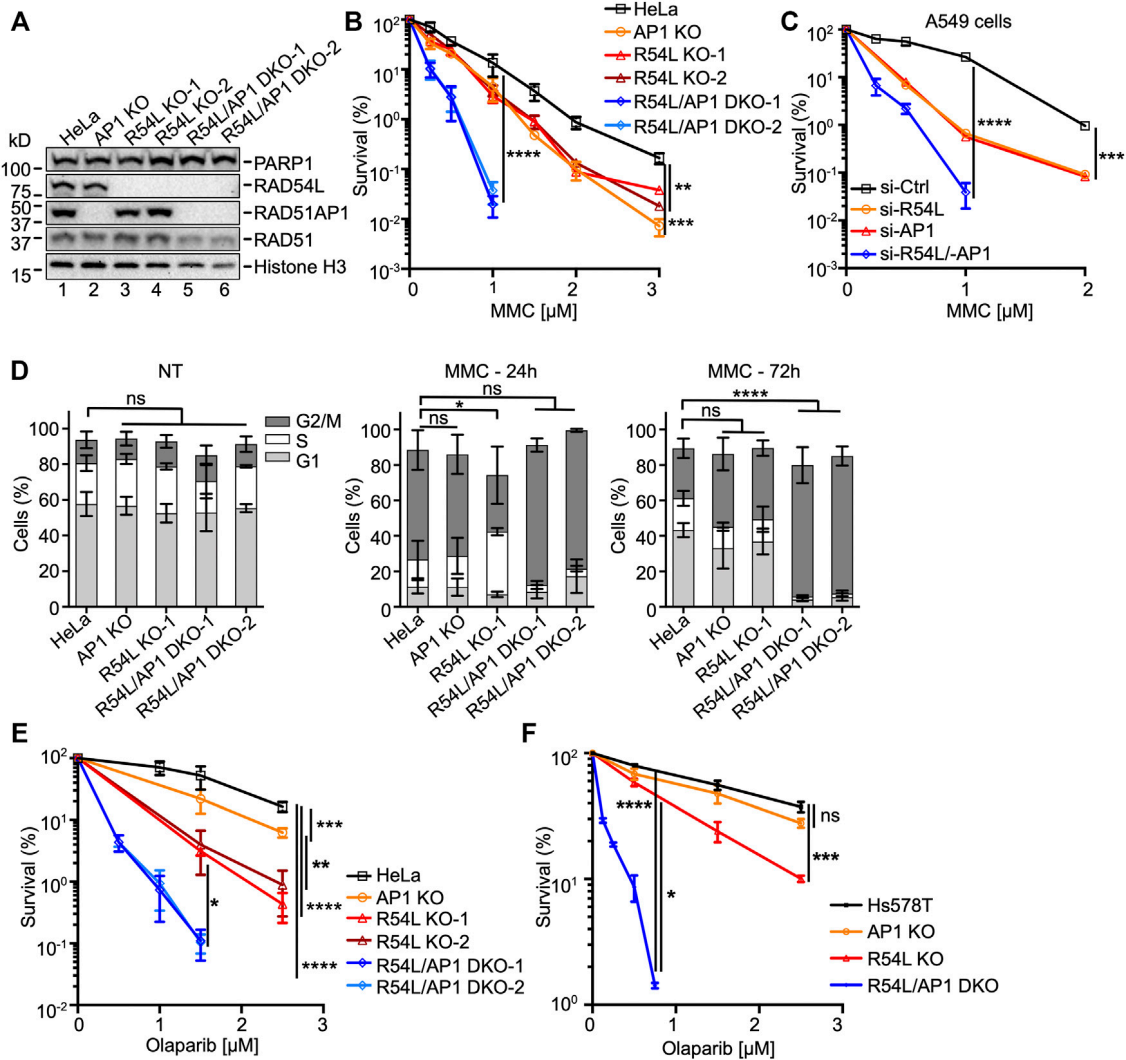
Deficiency of both *RAD51AP1* and *RAD54L* exacerbates MMC cytotoxicity and cell cycle progression, and sensitivity to olaparib. **(A)** Western blots of nuclear extracts of HeLa cells and derivatives. *RAD51AP1* KO cells (here: AP1 KO), two independently isolated *RAD54L* KO cell lines (here: R54L KO-1, R54L KO-2), and two independently isolated *RAD54L*/*RAD51AP1* DKO cell lines (here: R54L/AP1 DKO-1; R54L/AP1 DKO-2). Loading controls: PARP1, histone H3. **(B)** Results from clonogenic cell survival assays after MMC. Data points are the means from two to five independent experiments ±SD. **, *p* < 0.01; ***, *p* < 0.001; ****, *p* < 0.0001; two-way ANOVA followed by Tukey’s multiple comparisons test. **(C)** Results from clonogenic cell survival assays of MMC-treated A549 cells with RAD51AP1 and/or RAD54L knockdown. Data points are the means from three technical replicates for A549 cells transfected with RAD54L siRNA (here: si-R54L) or RAD51AP1 siRNA (si-AP1), and from two independent experiments ±SD for A549 cells transfected with negative control siRNA (si-Ctrl) or RAD54L and RAD51AP1 siRNA (si-R54L/-AP1). ***, *p* < 0.001; ****, *p* < 0.0001; two-way ANOVA followed by Tukey’s multiple comparisons test. **(D)** Average percentage of cells in G1, S and G2/M cell cycle phases without (here: NT (no treatment)), and 24 and 72 h after release from MMC. Bars are the means from at least three independent experiments ±SD. *, *p* < 0.05; ****, *p* < 0.0001; ns, non-significant; one-way ANOVA followed by Dunnett’s multiple comparisons test. **(E)** Results from olaparib clonogenic cell survival assays of HeLa, AP1 KO, R54L KO-1, R54L KO-2, and R54L/AP1 DKO-1 and DKO-2 cells. Data points are the means from three independent experiments ±SD. **(F)** Results from olaparib clonogenic cell survival assays of Hs578T, AP1 KO, R54L, and R54L/AP1 DKO cells. Data points are the means from three independent experiments ±SD. *, *p* < 0.05; **, *p* < 0.01; ***, *p* < 0.001; ****, *p* < 0.0001; ns, non-significant; two-way ANOVA followed by Tukey’s multiple comparisons test.

To investigate the genetic interaction between *RAD51AP1* and *RAD54L*, we generated *RAD54L*/*RAD51AP1* double knockout (DKO) HeLa cell lines and compared the phenotypes of these DKO cells to HeLa cells deleted for either *RAD51AP1* or *RAD54L* (Liang et al., 2019; Maranon et al., 2020). To generate *RAD54L*/*RAD51AP1* DKO cells we targeted *RAD54L* by CRISPR/Cas9-nic in *RAD51AP1* KO cells and selected two of several *RAD54L*/*RAD51AP1* DKO clones for the experiments described below. We verified the loss of protein expression by Western blot analysis (Figure 1A, lanes 5–6). PCR was performed across exon 8, and amplicons were sequenced across the Cas9-nic cleavage sites in *RAD54L* to confirm mutagenesis (Supplementary Figure S1A; Supplementary Tables S1, S6). Immunocytochemistry was used to monitor the loss of RAD54L foci formation after γ-irradiation (Supplementary Figure S1B).

We determined the growth rates of all HeLa cell derivatives (i.e., single KO and DKO cells) and detected no significant differences in population doubling times (Supplementary Figure S1C). In fractionated protein extracts from unperturbed cells, we noted higher levels of RAD54L protein in *RAD51AP1* KO cells (Supplementary Figure S1D, lanes 2 and 8) and higher levels of RAD51AP1 protein in *RAD54L* KO cells (Supplementary Figure S1D, lanes 9–10).

Next, we tested the sensitivity to MMC of single KO and DKO cells in clonogenic cell survival assays. In accord with published results by us and others (Liang et al., 2019; Maranon et al., 2020; Olivieri et al., 2020), we show that *RAD51AP1* and *RAD54L* single KO cells are moderately sensitized to the cytotoxic effects of MMC (Figure 1B). Deletion of both *RAD51AP1* and *RAD54L*, however, further sensitized HeLa cells to MMC (Figure 1B; Supplementary Table S2), in support of a non-epistatic relation between RAD51AP1 and RAD54L. To exclude that this effect was specific to HeLa cells, we depleted RAD51AP1 and/or RAD54L in A549 lung cancer cells (Supplementary Figure S1E). A549 cells depleted for either RAD51AP1 or RAD54L showed similarly increased sensitivities to MMC, while loss of both RAD51AP1 and RAD54L synergistically sensitized A549 cells to MMC (Figure 1C; Supplementary Table S2). Collectively, these results reveal compensation between RAD51AP1 and RAD54L for the protection of human cancer cell lines from MMC-induced DNA damage.

We used U2OS-DRGFP cells (Nakanishi et al., 2005; Xia et al., 2006) to assess the effects of RAD51AP1 and/or RAD54L depletion on gene conversion. Depletion of both RAD51AP1 and RAD54L downregulated the levels of gene conversion at DRGFP ∼10-fold (*p* < 0.001; Supplementary Figures S1F,G), while single knockdown of either RAD51AP1 or RAD54L impaired gene conversion ∼2-fold, as previously shown (Wiese et al., 2007; Spies et al., 2016).

Next, we assessed cell cycle progression upon MMC exposure of single and *RAD54L*/*RAD51AP1* DKO cells and compared the results to HeLa cells. In the absence of MMC, all cell lines progressed similarly through the cell cycle (Figure 1D, left panel, and Supplementary Figure S1H). Twenty-four hours after release from MMC, all cell lines remained arrested in cell cycle progression (Figure 1D, middle panel, and Supplementary Figure S1H). At 72 h post release from MMC, HeLa cells, *RAD51AP1* KO, and *RAD54L* KO cells regained the capacity to proceed through mitosis and enter the following cell cycle. Both *RAD54L/RAD51AP1* DKO cell lines, however, remained arrested in G2/M phase (*p* < 0.0001; Figure 1D, right panel, and Supplementary Figure S1H), likely due to their higher fraction of unresolved or mis-repaired DNA damage.

HR deficiency selectively confers sensitivity to PARPi (Bryant et al., 2005; Farmer et al., 2005). Hence, we asked if single KO and *RAD54L*/*RAD51AP1* DKO cells were sensitive to treatment with the PARPi olaparib. Compared to HeLa cells, *RAD51AP1* KO cells showed increased sensitivity to olaparib (*p* < 0.001; Figure 1E), as expected from earlier studies (Liang et al., 2019; Olivieri et al., 2020). The two *RAD54L* KO cell lines were more sensitive (*p* < 0.05 and *p* < 0.01 for KO-1 and KO-2 compared to *RAD51AP1* KO cells, respectively), and combined loss of both *RAD51AP1* and *RAD54L* synergistically sensitized HeLa cells to olaparib (*p* < 0.05 for *RAD54L*/*RAD51AP1* DKO-1 and DKO-2 compared to the *RAD54L* KO cells; Supplementary Table S2). To exclude that this effect was specific to HeLa cells, we generated *RAD54L*/*RAD51AP1* single KO and DKO cells in the Hs578T breast cancer cell line [(Hackett et al., 1977); Supplementary Figure S1I; Supplementary Table S1] and tested these cells in olaparib cell survival assays. As in HeLa cells, *RAD54L* single KO Hs578T cells showed significantly increased sensitivity to olaparib (*p* < 0.001), and combined loss of both *RAD51AP1* and *RAD54L* synergistically sensitized Hs578T cells to olaparib exposure (*p* < 0.05 for *RAD54L*/*RAD51AP1* DKO cells compared to *RAD54L* KO cells; Figure 1F; Supplementary Table S2). These results demonstrate compensatory activities between RAD51AP1 and RAD54L in protecting human cancer cell lines from olaparib-induced DNA damage.

### RAD54L Deficiency Is Rescued by Ectopic RAD54L

**FIGURE 2:**
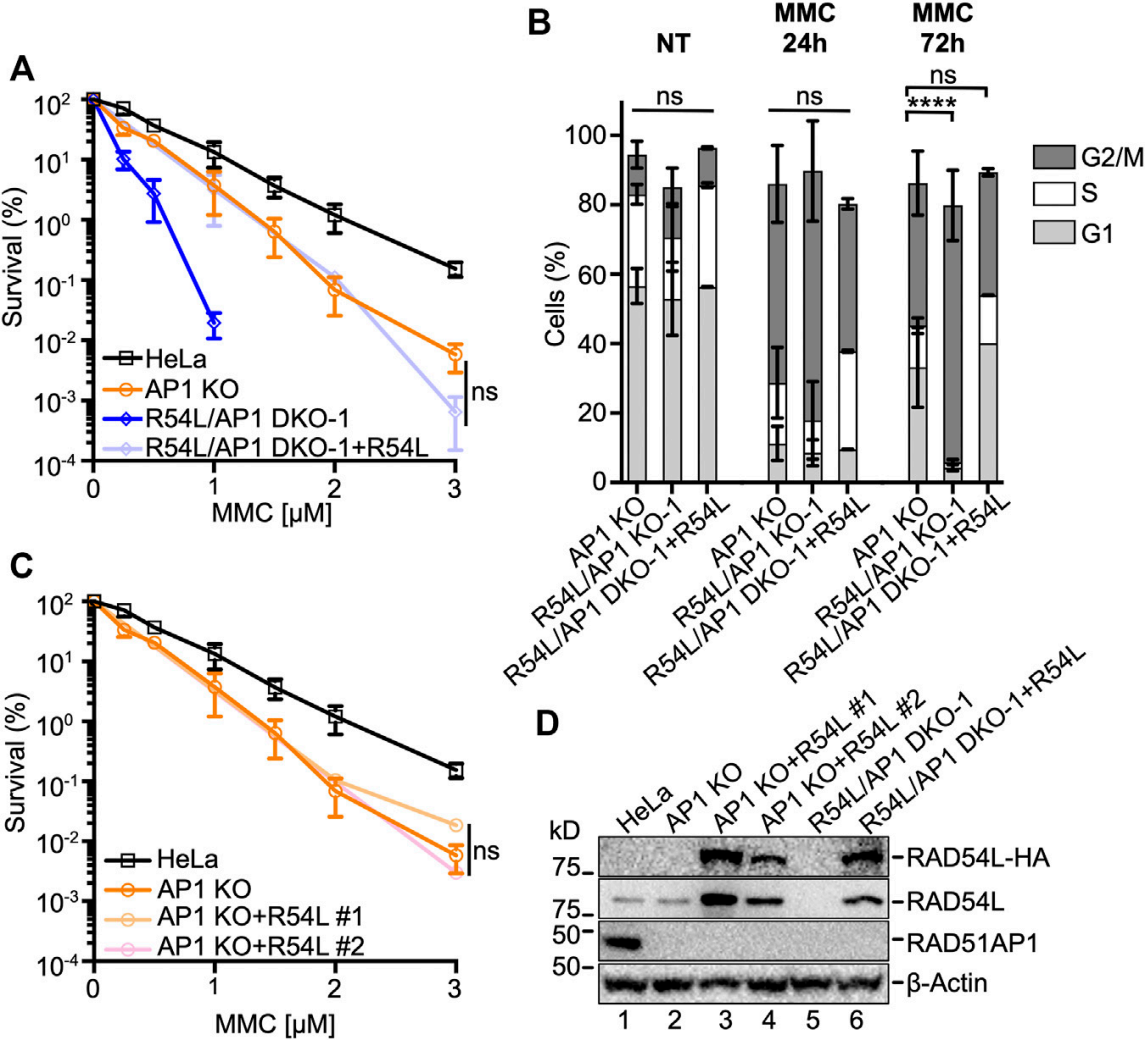
Ectopic expression of RAD54L rescues *RAD54L* deficiency in *RAD54L*/*RAD51AP1* double KO cells, but does not alter the response of *RAD51AP1* KO cells to MMC. **(A)** Results from MMC clonogenic cell survival assays of R54L/AP1 DKO-1 with (here: +R54L) and without ectopic RAD54L and of AP1 KO and HeLa cells for comparison purposes. Data points are the means from two independent experiments ±SD. ns, non-significant; two-way ANOVA followed by Tukey’s multiple comparisons test. **(B)** Average percentage R54L/AP1 DKO-1 cells with (here: +R54L) and without ectopic RAD54L and of AP1 KO cells (same data as in Figure 1D) in G1, S and G2/M cell cycle phases without MMC (NT), and 24 and 72 h after release from MMC. Bars represent the means from two independent experiments ±SD. ****, *p* < 0.0001; ns, non-significant; one-way ANOVA followed by Dunnett’s multiple comparisons test. **(C)** Results from MMC clonogenic cell survival assays of AP1 KO cells and two independently isolated AP1 KO clones expressing different amounts of ectopic RAD54L **(D)**. Data points are the means from two independent experiments ±SD for AP1 KO + R54L #1 cells and from three technical replicates for AP1 KO + R54L #2 cells. ns, non-significant; two-way ANOVA followed by Tukey’s multiple comparisons test. **(D)** Western blots of whole cell protein extracts to show stably expressed ectopic RAD54L-HA (here: +R54L) in AP1 KO cells (lanes 3 and 4) and in R54L/AP1 DKO cells (lane 6). Loading control: β-Actin.

Ectopic expression of HA-tagged RAD54L in *RAD54L*/*RAD51AP1* DKO HeLa cells reverted their response to MMC to the level of *RAD51AP1* KO cells (Figures 2A,D). As expected, cell cycle progression after MMC of *RAD54L*/*RAD51AP1* DKO cells with ectopic RAD54L became similar to that of *RAD51AP1* KO cells (Figure 2B; Supplementary Figure S1H). Moreover, *RAD54L*/*RAD51AP1* double KO cells with ectopic RAD54L formed RAD54L foci after γ-irradiation (Supplementary Figure S1B). Ectopic expression of RAD54L also rescued the sensitivity to MMC of single *RAD54L* KO cells (Supplementary Figures S2A,B) and RAD54L foci formation after γ-irradiation (Supplementary Figure S1B). These results show that the phenotypes associated with *RAD54L* deficiency in *RAD54L*/*RAD51AP1* DKO and *RAD54L* single KO cells stem from the loss of *RAD54L*. However, ectopic expression of high amounts of RAD54L in *RAD51AP1* KO cells (Figure 2D, lanes 3–4) did not rescue their sensitivity to MMC (Figure 2C), demonstrating that defined attributes of the RAD51AP1 protein cannot be compensated for by RAD54L.

### Deletion of Both *RAD51AP1* and *RAD54L* Sensitizes HeLa Cells to Replication Stress

**FIGURE 3:**
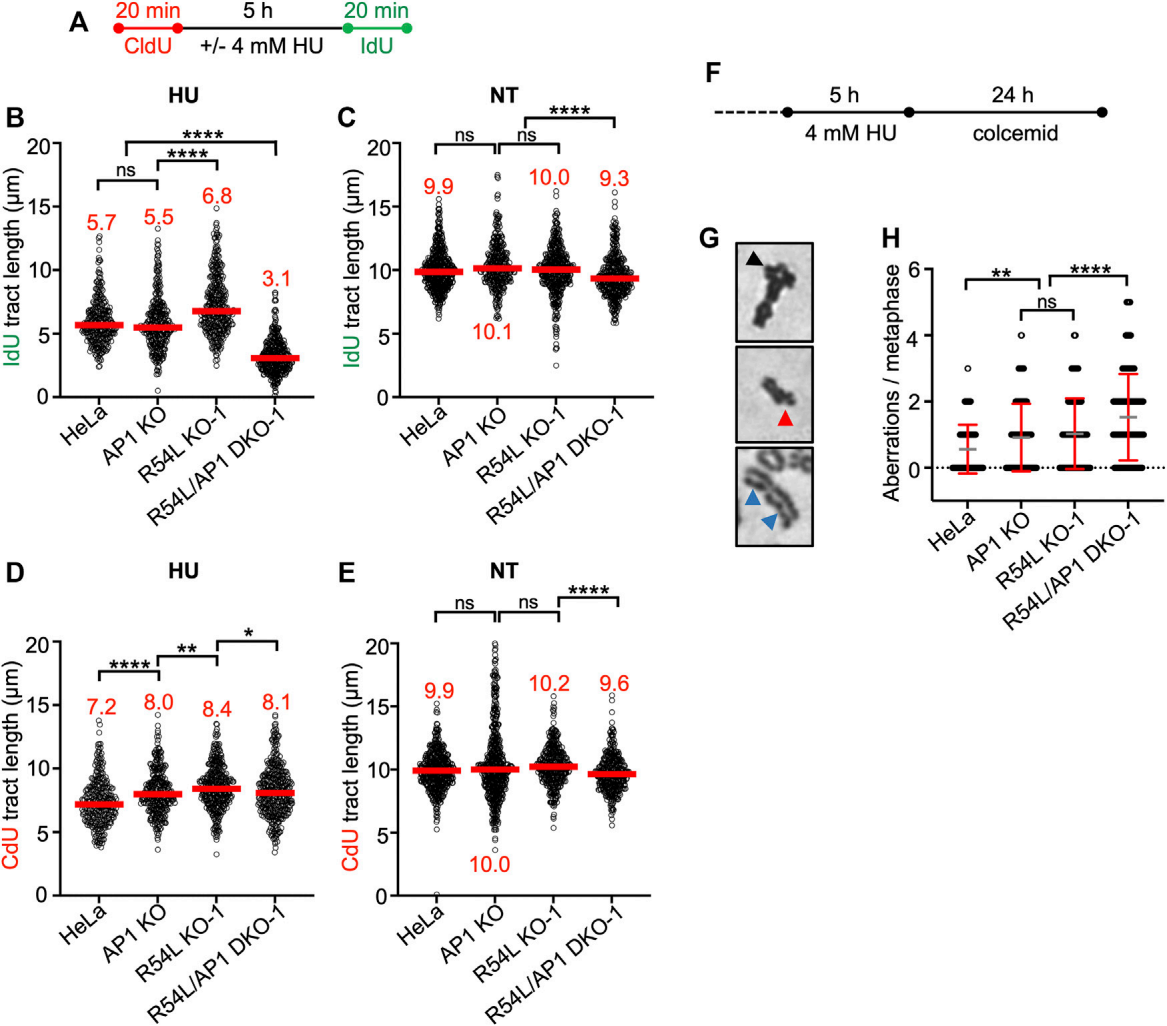
Concomitant loss of *RAD51AP1* and *RAD54L* results in increased replication stress and genome instability. **(A)** Schematic of the experimental protocol for the DNA fiber assay. **(B)** Median IdU tract length (green) after HU in HeLa, AP1 KO, R54L KO-1, and R54L/AP1 DKO-1 cells. Data points are from 100 to 150 fibers of three independent experiments each, with medians in red. **(C)** Median IdU tract length (green) under unperturbed conditions (NT). Data points are from 100 to 150 fibers of three independent experiments each, with medians in red. **(D)** Median CldU tract length (red) after HU in HeLa, AP1 KO, R54L KO-1, and R54L/AP1 DKO-1 cells. Data points are from 100 to 150 fibers of three independent experiments each, with medians in red. **(E)** Median CdU tract length (green) under unperturbed conditions (NT). Data points are from 100 to 150 fibers of three independent experiments each, with medians in red. *, *p* < 0.05; **, *p* < 0.01; ****, *p* < 0.0001; ns, non-significant; Kruskal-Wallis test followed by Dunn’s multiple comparisons test. **(F)** Schematic of the experimental protocol to induce chromosomal aberrations. **(G)** Representative micrographs of chromosomal aberrations after HU; radial (black arrow head), chromatid break (red arrow head) and chromatid gaps (blue arrow heads). **(H)** Aberrations per metaphase after HU in HeLa, AP1 KO, R54L KO-1, and R54L/AP1 DKO-1 cells. Data points are from 100 metaphases of two independent experiments each. Means (grey lines) ± SD (red lines) are shown. **, *p* < 0.01; ****, *p* < 0.0001; ns, non-significant; one-way ANOVA followed by Tukey’s multiple comparisons test.

We treated all HeLa cell derivatives with 4 mM HU for 5 h, which blocks DNA synthesis and stalls replication fork movement (Liu et al., 2020). To understand the fate of stalled replication forks in single KO and DKO cells, we monitored the recovery of cells from stalled replication using the single-molecule DNA fiber assay. We pulse-labeled cells with the thymidine analog 5-Chloro-2′-deoxyuridine (CldU) first, then replenished cells with HU-containing medium prior to pulse-labeling with 5-Iodo-2′-deoxyuridine (IdU) (Figure 3A). We determined the ability of all cell lines to restart DNA replication by measuring the lengths of IdU tracts of CldU-labeled DNA fibers (Figure 3B; Supplementary Figure S3B; Supplementary Table S3). *RAD51AP1* KO cells showed no significant defect in fork restart compared to HeLa cells. In contrast, *RAD54L* KO cells restarted forks significantly faster than HeLa cells (*p* < 0.0001), possibly related to the role of RAD54L in catalyzing fork regression (Bugreev et al., 2011). Interestingly, in comparison to both HeLa and single KO cells, *RAD54L*/*RAD51AP1* DKO cells were significantly impaired in fork restart (*p* < 0.0001; Figure 3B; Supplementary Table S3). Collectively, these results show that the efficient restart from stalled DNA replication relies on RAD54L in *RAD51AP1* KO HeLa cells. The results also suggest that the RAD54L protein suppresses accelerated fork restart after HU, an attribute not shared by RAD51AP1.

In unperturbed cells, DNA replication progressed slower in *RAD54L*/*RAD51AP1* DKO cells than in HeLa cells or the single KOs, suggesting that endogenous obstacles to fork progression may impede replication in the absence of both RAD54L and RAD51AP1 (*p* < 0.0001; Figure 3C; Supplementary Table S3).

In response to replication stress, replication forks reverse into four-way junctions through annealing of the nascent DNA strands (Zellweger et al., 2015). Fork reversal is mediated by RAD51 and several DNA motor proteins and serves to bypass obstacles encountered by the replisome (Mijic et al., 2017; Thakar and Moldovan, 2021; Thangavel et al., 2015; Zellweger et al., 2015). Reversed forks must be protected from nucleolytic attack to prevent fork attrition (Lemacon et al., 2017; Petermann et al., 2010; Schlacher et al., 2011; Taglialatela et al., 2017; Thangavel et al., 2015). To assess if RAD54L and/or RAD51AP1 function in the protection of replication forks from unprogrammed nuclease degradation, CldU tracts in cells exposed to HU were measured and compared to the CldU tract lengths in untreated cells. CldU tracts after HU were shorter than those in unperturbed cells for all cell lines tested (Figures 3D,E; Supplementary Table S3). Overall, however, CldU tracts in HU-treated *RAD51AP1* KO, *RAD54L* KO, and *RAD54*/*RAD51AP1* DKO cells were not shorter than those in HU-treated HeLa cells (Figure 3D; Supplementary Figure S3B; Supplementary Table S3). These results suggest that RAD54L and RAD51AP1 largely function independently of the protection mechanism of reversed forks in overcoming replication stress in HeLa cells. We infer that replication forks in HeLa and KO cells are degraded as part of the normal cellular physiology in response to prolonged fork stalling by HU (Thangavel et al., 2015), and that the recruitment of proteins involved in the protection of nascent DNA at replication forks likely proceeds normally in *RAD54L*/*RAD51AP1* single KO and DKO cells.

### Concomitant Loss of *RAD51AP1* and *RAD54L* Exacerbates Genome Instability

Next, we tested the consequences of HU-induced replication stress to cells with impaired replication restart. To this end, we determined chromatid gaps and breaks, and complex chromosome aberrations (i.e., radials) in HeLa, single KO and *RAD54L*/*RAD51AP1* DKO cells after treatment with HU (Figures 3F–H). Exposure to HU led to 0.56 ± 0.73 mean aberrations per metaphase in HeLa cells and to a significant increase in mean aberrations per metaphase in both *RAD51AP1* (0.91 ± 1.02; *p* < 0.01) and *RAD54L* single KO cells (1.03 ± 1.07; *p* < 0.0001; Figure 3H). As expected, in *RAD54L*/*RAD51AP1* DKO cells, the mean number of aberrations per metaphase was further increased compared to the single KOs (1.53 ± 1.30; *p* < 0.0001). These results show that replication stress leads to genome instability most prominently in *RAD54L*/*RAD51AP1* DKO cells, which also show the most pronounced defect in fork restart.

### RAD54B Compensates for RAD54L in the Presence of RAD51AP1

**FIGURE 4:**
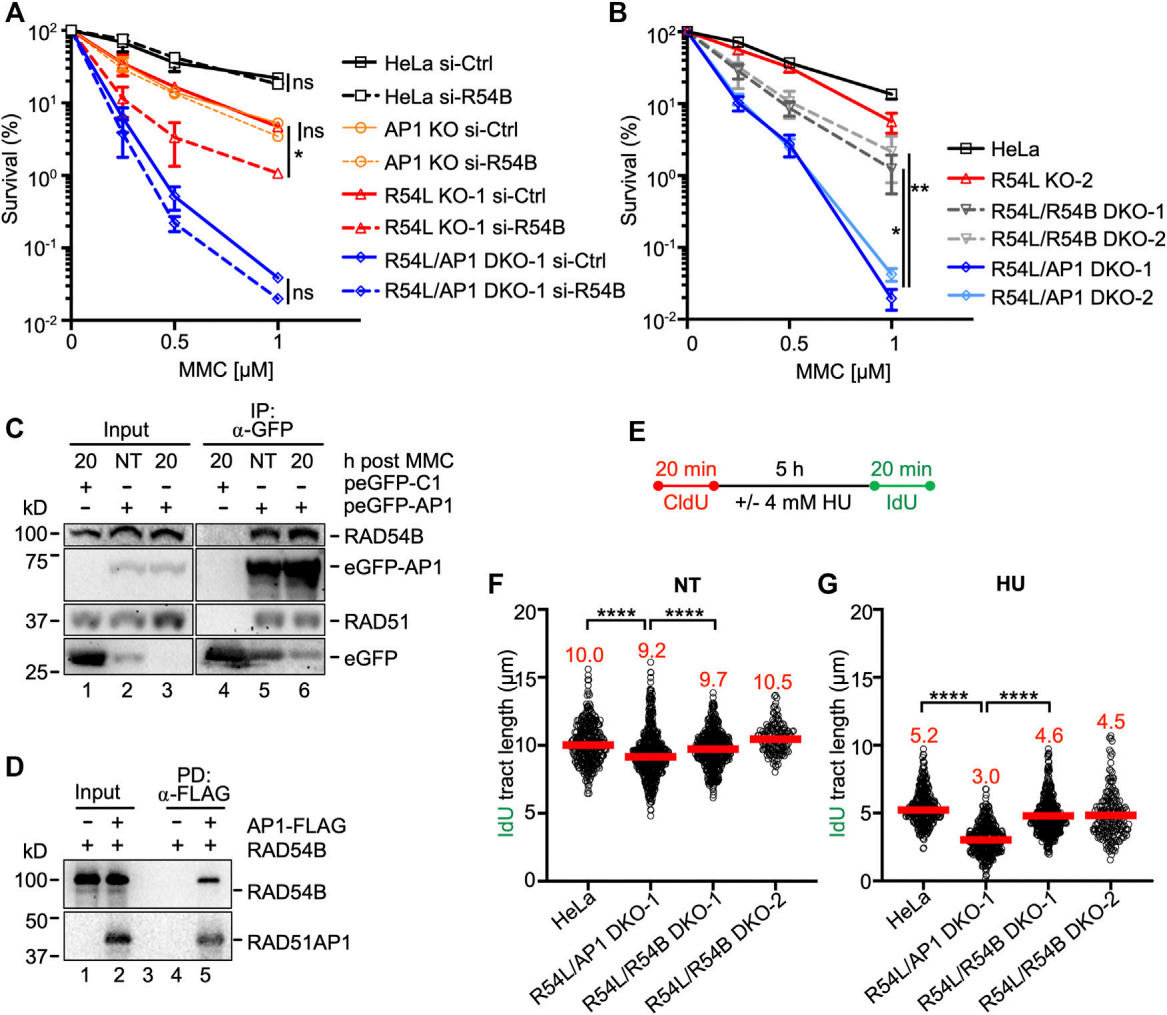
Concomitant loss of *RAD54L* and *RAD54B* exacerbates MMC cytotoxicity and replication stress less severeley than concomitant loss of *RAD54L* and *RAD51AP1*. **(A)** Results from clonogenic cell survival assays after MMC of cells transfected with negative control (si-Ctrl) or RAD54B siRNA (si-R54B): HeLa, AP1 KO, R54L KO-1, R54L/AP1 DKO-1 cells. Data points are the means from two independent experiments ±SD. *, *p* < 0.05; ns, non-significant; two-way ANOVA followed by Tukey’s multiple comparisons test. **(B)** Results from clonogenic cell survival assays after MMC treatment of HeLa, R54L KO-2, R54L/54B DKO-1 and KO-2, and R54L/AP1 DKO-1 and DKO-2 cells. Data points are the means from two independent experiments ±SD. *, *p* < 0.05; **, *p* < 0.01; two-way ANOVA followed by Tukey’s multiple comparisons test. **(C)** Western blots to show that endogenous RAD54B co-precipitates in anti-eGFP protein complexes of R54L/AP1 DKO cells ectopically expressing eGFP-RAD51AP1 (here: peGFP-AP1) in the absence of MMC (NT; lane 5) and 20 h after a 2-h incubation in 0.5 μM MMC (lane 6). RAD51: positive control for interaction, as previously shown in different cell types (Kovalenko et al., 1997; Wiese et al., 2007). Lane 4: Neither RAD54B nor RAD51 co-precipitate in anti-eGFP protein complexes generated from R54L/AP1 DKO cells transfected with control plasmid (peGFP-C1). **(D)** Western blots to show direct interaction between purified FLAG-tagged RAD51AP1 protein (here: AP1-FLAG) and purified RAD54B precipitated by anti-FLAG M2 affinity resin (lane 5). **(E)** Schematic of the protocol for the DNA fiber assay. **(F)** Median IdU tract length under unperturbed conditions (NT) in HeLa, R54L/AP1 KO-1, and R54L/R54B DKO-1 and DKO-2 cells. Data points are from 150 to 200 fibers of three experiments for HeLa, R54L/AP1 DKO-1 and R54L/R54B DKO-1 cells and from one experiment for R54L/R54B DKO-2 cells, with medians in red. **(G)** Median IdU tract length after HU in HeLa, R54L/AP1 KO-1, and R54L/R54B DKO-1 and DKO-2 cells. Data points are from 150 to 200 fibers of three experiments for HeLa, R54L/AP1 DKO-1 and R54L/R54B DKO-1 cells and from one experiment for R54L/R54B DKO-2 cells, with medians in red. ****, *p* < 0.0001; Kruskal-Wallis test followed by Dunn’s multiple comparisons test.

In human cells, the role of RAD54B in HR is not well understood. In mice, however, the contribution of RAD54B to HR was discovered in the absence of RAD54L (Wesoly et al., 2006). To investigate the impact of RAD54B on the protection of human cells from MMC-induced DNA damage, we depleted RAD54B in HeLa, single KO, and *RAD54L*/*RAD51AP1* DKO cells (Supplementary Figure S4A) and performed MMC cell survival assays. Depletion of RAD54B in HeLa and *RAD51AP1* KO cells had no effect on their sensitivity to MMC (Figure 4A). Similarly, depletion of RAD54B in *RAD54L*/*RAD51AP1* DKO cells did not increase MMC cytotoxicity. In contrast, depletion of RAD54B in *RAD54L* KO cells further sensitized *RAD54L* KO cells to MMC (*p* = 0.044; Figure 4A; Supplementary Table S2). These results show that the activity of RAD54B is critical for the protection of human cells from MMC cytotoxicity in the absence of RAD54L. In HeLa, *RAD51AP1* KO, and *RAD54L*/*RAD51AP1* DKO cells, however, RAD54B appears to play no detectable role in the protection of cells from MMC-induced DNA damage.

To exclude the possibility that the mild increase in MMC sensitivity of *RAD54L* KO cells depleted for RAD54B was the result of incomplete RAD54B knockdown, we generated *RAD54L*/*RAD54B* DKO HeLa cells (Supplementary Figure S4B; Supplementary Table S1) and compared their response to MMC to that of the *RAD54L*/*RAD51AP1* DKOs. As observed after RAD54B knockdown, two independently isolated *RAD54L*/*RAD54B* DKO cells lines were significantly more resistant to MMC than *RAD54L*/*RAD51AP1* DKO cells (*p* = 0.037 and *p* = 0.007 for *RAD54*/*RAD54B* KO-1 and KO-2, respectively; Figure 4B). These results show that in the absence of RAD54L, human cells more heavily rely on RAD51AP1 than on RAD54B to resist MMC cytotoxicity.

### RAD54B Co-Precipitates in RAD51AP1 Complexes

Since knockdown of RAD54B did not further increase the sensitivity to MMC of *RAD51AP1* single KO and *RAD54L/RAD51AP1* DKO cells, we hypothesized that this—in part—could be the result of RAD54B and RAD51AP1 acting in unity during the protection of cells from MMC-induced cytotoxicity. As such, we asked if RAD51AP1 may function in conjunction with RAD54B in human cells, and if a complex between these two proteins could be identified. Using the purified proteins, we previously showed that RAD51AP1 and RAD54L physically interact, and that both proteins compete in binding to RAD51 (Maranon et al., 2020). Based on these results, we first tested if endogenous RAD54L would co-precipitate in anti-RAD51AP1 complexes of *RAD51AP1* KO cells stably expressing FLAG-tagged RAD51AP1. Our results show that RAD54L co-precipitates with FLAG-RAD51AP1 under unperturbed conditions (Supplementary Figure S4C, lane 4).

Next, we tested the association between RAD51AP1 and RAD54B in human cells. As RAD54B activity is more prevalent in the absence of RAD54L (Figures 4A,B), we used *RAD54L*/*RAD51AP1* DKO cells with transiently expressed eGFP-tagged RAD51AP1. Both RAD51 and RAD54B were present in anti-eGFP precipitates from *RAD54L*/*RAD51AP1* DKO cells expressing eGFP-RAD51AP1 (Supplementary Figure S4D, lane 7); in contrast, RAD54B was absent in anti-eGFP precipitates from *RAD54L*/*RAD54B* DKO cells expressing eGFP-RAD51AP1 (Supplementary Figure S4D, lane 8). We then prepared protein lysates from *RAD54L*/*RAD51AP1* DKO cells transiently expressing eGFP-RAD51AP1 under unperturbed conditions (NT), and at 4 and 20 h after release from a 2-h treatment with 0.5 µM MMC. RAD54B was present in anti-eGFP complexes from both untreated and MMC-treated cells (Figure 4C, lanes 5–6, and Supplementary Figure S4E, lanes 7–8). These results show that endogenous RAD54B can associate with ectopically expressed RAD51AP1 in *RAD54L/RAD51AP1* DKO cells in the absence and in the presence of MMC-induced DNA damage.

To determine if RAD54B and RAD51AP1 physically interact, we performed a FLAG pull-down assay with the purified proteins (Supplementary Figure S4F). RAD54B co-precipitated with RAD51AP1-FLAG on anti-FLAG beads (Figure 4D, lane 5), indicating that RAD54B directly interacts with RAD51AP1.

### Deletion of Both RAD54L and RAD54B Sensitizes HeLa Cells to Replication Stress

To understand the consequences of concomitant RAD54L and RAD54B loss on replication fork dynamics, we used the DNA fiber assay, as described above (for schematic of the protocol see Figure 4E). As shown in Figure 3C and herein determined independently, replication progressed significantly more slowly in *RAD54L*/*RAD51AP1* DKO cells than in HeLa cells under unperturbed conditions (*p* < 0.0001; Figure 4F; Supplementary Figure S3B; Supplementary Table S3). Fork progression in unperturbed *RAD54L*/*RAD54B* DKO-1 and DKO-2 cells was faster than in *RAD54L*/*RAD51AP1* DKO cells (Figure 4F; Supplementary Table S3). After HU, fork restart was significantly slower in *RAD54L*/*RAD51AP1* DKO cells than in *RAD54L*/*RAD54B* DKO cells (*p* < 0.0001; Figure 4G; Supplementary Table S3). These results show that, in response to stalled DNA replication in the absence of RAD54L, the activities of both RAD51AP1 and RAD54B are important to efficiently restart replication forks. However, concomitant loss of RAD54L and RAD51AP1 is more detrimental to the recovery from stalled replication than concomitant loss of RAD54L and RAD54B.

As observed earlier (Figure 3D), CldU tracts after HU in HeLa and *RAD54L*/*RAD51AP1* DKO cells were shorter than in unperturbed cells (Supplementary Figures S4G,H; Supplementary Table S3). In the *RAD54L*/*RAD54B* DKOs, however, CldU tract lengths were not affected by treatment of cells with HU (*p* = 0.635 (Mann-Whitney test); Supplementary Figures S4G,H; Supplementary Table S3), suggesting that, in response to prolonged fork stalling by HU, *RAD54L*/*RAD54B* DKO HeLa cells are less sensitive to fork degradation.

### Deletion of *RAD54B* in *RAD54L* KO Cells Further Sensitizes HeLa Cells to Olaparib

**FIGURE 5:**
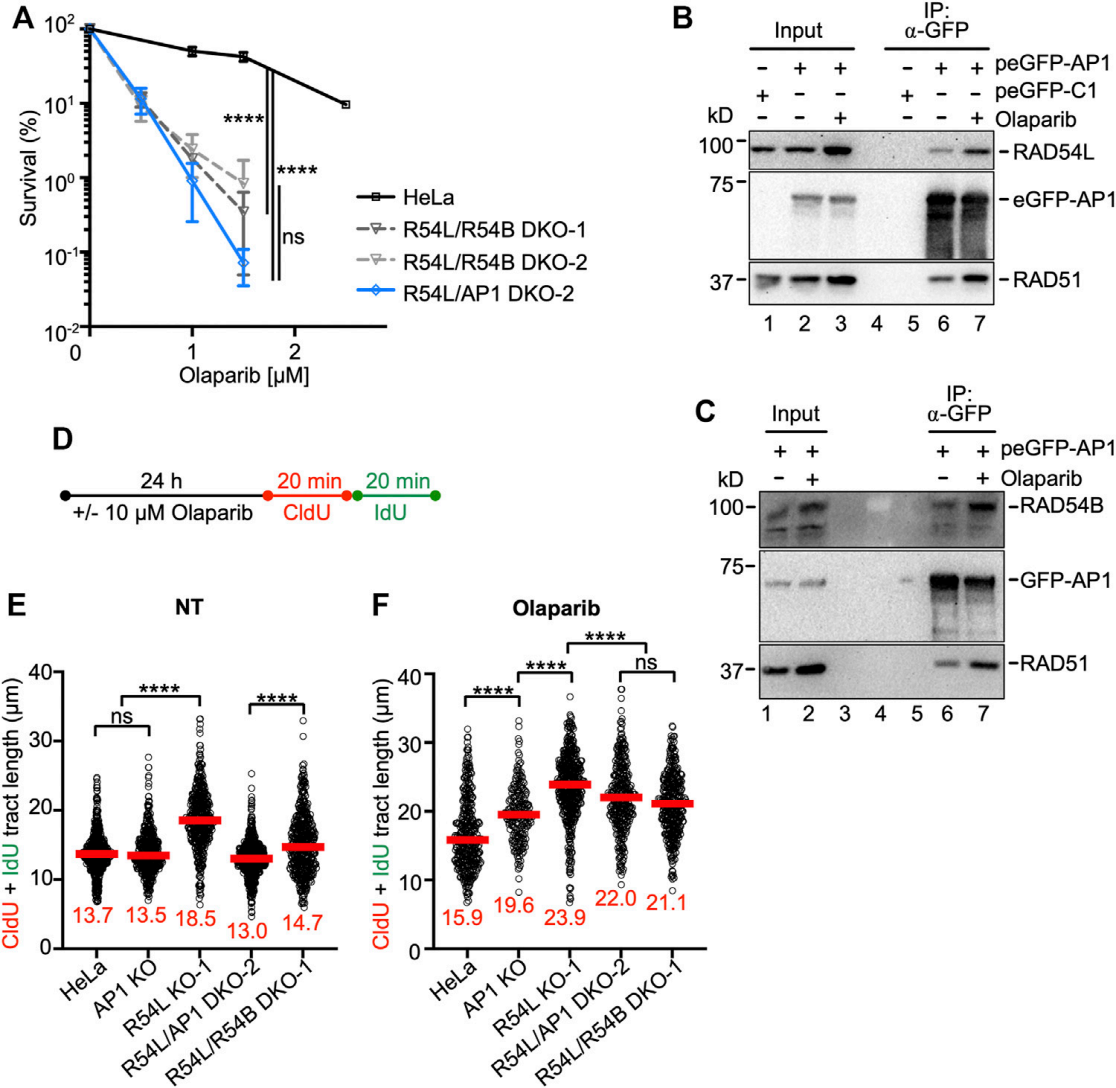
Loss of *RAD54L* and *RAD51AP1* or of *RAD54L* and *RAD54B* enhances cellular sensitivity to olaparib to similar extent. **(A)** Results from olaparib clonogenic cell survival assays of HeLa, R54L/R54B DKO-1 and DKO-2, and R54L/AP1 DKO-2 cells. Data points are the means from two independent experiments ±SD. ****, *p* < 0.0001; ns, non-significant; two-way ANOVA followed by Tukey’s multiple comparisons test. **(B)** Western blots to show that endogenous RAD54L co-precipitates in anti-eGFP protein complexes of AP1 KO cells ectopically expressing eGFP-RAD51AP1 (here: peGFP-AP1) in the absence (lane 6) and after a 1-day incubation in 10 μM olaparib (lane 7). RAD51: positive control for interaction, as previously shown in different cell types (Kovalenko et al., 1997; Wiese et al., 2007). Lane 5: negative control (cells transfected with peGFP-C1 vector). **(C)** Western blots to show that endogenous RAD54B co-precipitates in anti-eGFP protein complexes of R54L/AP1 DKO cells ectopically expressing eGFP-RAD51AP1 (here: peGFP-AP1) in the absence (lane 6) and after a 1-day incubation in 10 μM olaparib (lane 7). **(D)** Schematic of the experimental protocol for the DNA fiber assay in unperturbed cells and after olaparib. **(E,F)** Median tract length (CldU + IdU) of DNA fibers in HeLa, AP1 KO, R54L KO-1, R54L/AP1 DKO-2 and R54L/R54B DKO-1 without (NT) and with olaparib treatment. Data points are from 130 to 230 fibers of three independent experiments each, with medians in red. ****, *p* < 0.0001; ns, non-significant; Kruskal-Wallis test followed by Dunn’s multiple comparisons test.

Next, we compared the cytotoxicity of olaparib to *RAD54L/RAD51AP1* and *RAD54L/RAD54B* DKO cells. Surprisingly, treatment with olaparib decreased the survival of both *RAD54L*/*RAD51AP1* and *RAD54L*/*RAD54B* DKO cells to similar extent (*p* < 0.0001 compared to HeLa cells; Figure 5A). We also generated a *RAD51AP1*/*RAD54B* DKO cell line (Supplementary Figure S5A; Supplementary Table S3) and tested these cells for their sensitivity to olaparib. We found that *RAD51AP1* single KO cells and *RAD51AP1*/*RAD54B* DKO cells exhibit identical sensitivities to olaparib (Supplementary Figure S5B). Collectively, these results suggest that RAD51AP1 and RAD54B largely function within the same HR sub-pathway upon treatment of cells with olaparib. This sub-pathway compensates RAD54L deficiency.

Under unperturbed conditions and after a 1-day incubation of cells in 10 µM olaparib, endogenous RAD54L co-precipitated with transiently expressed eGFP-RAD51AP1 in *RAD51AP1* KO cells (Figure 5B, lanes 6 and 7, respectively). Similarly, endogenous RAD54B co-precipitated with transiently expressed eGFP-RAD51AP1 in *RAD54L*/*RAD51AP1* DKO cells under unperturbed conditions and upon treatment of cells with olaparib (Figure 5C, lanes 6 and 7, respectively). These results show that RAD54L or RAD54B can be part of a larger protein complex involving RAD51AP1 and RAD51, and that for both RAD54L and RAD54B complex formation with RAD51AP1 is enhanced upon treatment of cells with olaparib.

We analyzed the dynamics of replication fork progression by DNA fiber assay after a 1-day incubation of cells in 10 µM olaparib (Figure 5D). Compared to untreated cells, fiber tracts were longer in HeLa cells after olaparib (Figures 5E,F; Supplementary Figure S5D; Supplementary Table S3), consistent with the results from an earlier study (Maya-Mendoza et al., 2018). After olaparib, in *RAD51AP1* and *RAD54L* single KO and in *RAD54L*/*RAD51AP1* and *RAD54L/RAD54B* DKO cells, fiber tracts were significantly longer than in HeLa cells (*p* < 0.0001; Figure 5F; Supplementary Table S3), indicative of the further increased defects of the KO cell lines in restraining fork progression. Compared to the lengths of fiber tracts obtained under unperturbed conditions (Figure 5E), median fiber tracts were 16% longer in HeLa cells, 45% longer in *RAD51AP1* KO cells, 29% longer in *RAD54L* KO cells and 69% and 44% longer in *RAD54L/RAD51AP1* and *RAD54L/RAD54B* DKO cells, respectively. Collectively, these results show that HR-proficient HeLa cells restrain accelerated fork elongation more effectively than any of the KO cell lines. Moreover, while a 1-day exposure to olaparib is associated with increased levels of DSBs in all cell lines investigated, COMET assays revealed significantly more DSBs in *RAD54L*/*RAD51AP1* and *RAD54*/*RAD54B* DKO cells than in HeLa cells and the single KOs (*p* < 0.0001; Supplementary Figure S5G; Supplementary Table S4). These results suggest that fork stability is particularly compromised when fork movement is accelerated in *RAD54L*/*RAD51AP1* and *RAD54L*/*RAD54B* DKO cells, and that the stress to replication forks, as determined by COMET assay, is similar in both DKOs.

## Discussion

**FIGURE 6:**
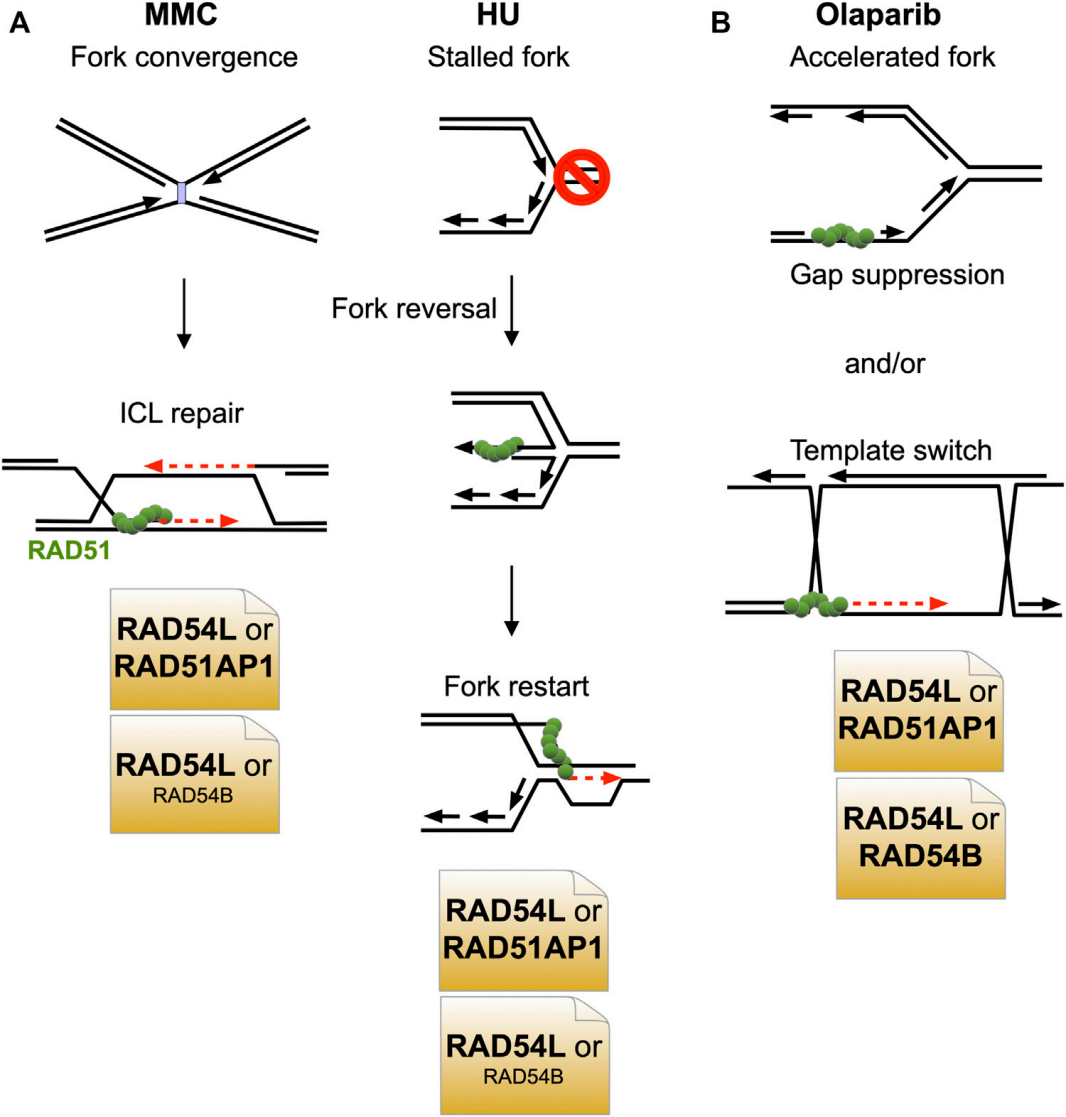
Model depicting the predominant negative genetic interactions between RAD54L and RAD51AP1 and between RAD54L and RAD54B. **(A)** Upon treatment of cells with MMC or HU, *RAD54L* loss is compensated for more extensively by RAD51AP1 than by RAD54B. **(B)** Upon treatment of cells with olaparib, RAD51AP1 and RAD54B compensate for the loss of *RAD54L* to similar extent.

In this study, we have shown that the HR function of RAD54L can largely be compensated for by the RAD51AP1 protein. Surprisingly, in the context of stalled and collapsed DNA replication (after HU or MMC), the compensatory activity of RAD51AP1 is greater than that of the RAD54L paralog RAD54B (Figure 6A). After treatment of cells with olaparib, however, RAD51AP1 and RAD54B are equally important in substituting for RAD54L (Figure 6B).

Given that RAD54B and RAD51AP1 physically interact (this study), evidence of physical interaction and functional cooperation between RAD54B and RAD51 (Sarai et al., 2006; Wesoly et al., 2006), and of an indirect association between human RAD54B and RAD51 in the context of chromatin and in cells (Tanaka et al., 2000; Zhang et al., 2007), it is possible that in select stages of the HR reaction, within a certain context of the genome, or in response to specific types of DNA damage, RAD51AP1 functions cooperatively with RAD54B, possibly bridging RAD54B to RAD51. As *RAD54L* KO cells with RAD54B knockdown show increased sensitivity to MMC but RAD54B-depleted *RAD54L*/*RAD51AP1* double KO cells do not, this may be further evidence of RAD54B functioning in conjuncture with RAD51AP1. Nonetheless, *RAD54L*/*RAD51AP1* double KO cells are significantly more sensitive to MMC than *RAD54L*/*RAD54B* double KO cells, which argues that RAD51AP1 has additional function(s) aside from working with RAD54B in repairing MMC-induced DNA damage. This could be together with another and yet to be identified translocase, or within transcriptionally active, decondensed regions of the genome in which RAD51AP1 was shown to promote HR (Ouyang et al., 2021).

In contrast, an only epistatic relation between RAD51AP1 and RAD54B appears to exist in upon treatment of cells with olaparib, suggesting that both proteins function within the same pathway in response to replication stress induced by faster fork progression (Maya-Mendoza et al., 2018). Olaparib leads to the formation of replication associated ssDNA gaps (Maya-Mendoza et al., 2018; Thakar and Moldovan, 2021). Gap suppression mechanisms and HR-mediated post-replicative repair serve to restrict and eliminate ssDNA replication gaps (Hashimoto et al., 2010; Piberger et al., 2020; Cong et al., 2021; Panzarino et al., 2021). Concomitant loss of RAD54L and either RAD51AP1 or RAD54B may exacerbate ssDNA gap formation and thereby inhibit nascent DNA strand annealing (Cong et al., 2021), resulting in the similar degree of Olaparib cytotoxicity in *RAD54L*/*RAD51AP1* and *RAD54L*/*RAD54B* DKO cells (Figure 6B).

RAD51AP1 expression is increased in different breast cancer subtypes and other cancers and inversely associated with overall survival (Song et al., 2004; Henson et al., 2006; Martin et al., 2007; Martinez et al., 2007; Obama et al., 2008; Pathania et al., 2016; Chudasama et al., 2018; Li et al., 2018; Bridges et al., 2020; Zhao et al., 2020; Zhuang et al., 2020). Moreover, *Rad51ap1* deficiency abrogates tumor growth and metastasis in a breast cancer mouse model (Bridges et al., 2020), suggesting that the RAD51AP1 protein may be a promising target of inhibition in anti-cancer therapy. Given our results showing extensive compensation between RAD51AP1 and RAD54L, we surmise that the simultaneous inactivation of both RAD51AP1 and RAD54L could be a viable strategy to treat cancer in the context of induced DNA damage. Targeting RAD51AP1 together with RAD54L may be particularly effective against tumors with overactive HR (Raderschall et al., 2002; Xu et al., 2005; Klein, 2008; Marsden et al., 2016), cancerous cells maintaining their telomeres by the ALT pathway (Barroso-Gonzalez et al., 2019; Mason-Osann et al., 2020; Recagni et al., 2020), and BRCA1/2-mutant tumors that have regained HR proficiency and are resistant to PARPi (Han et al., 2020; Kim et al., 2021).

## Materials and Methods

### Cell Culture, Transfection and siRNAs

HeLa and A549 cells were obtained from ATCC and were maintained as recommended. HeLa cells in which either *RAD51AP1* or *RAD54L* is deleted were maintained as described previously (Liang et al., 2019; Maranon et al., 2020). Hs578T cells were a gift from Dr. Joe Gray (OHSU) and maintained as described (Colston et al., 1998). The siRNAs used were described previously (Parplys et al., 2015; Maranon et al., 2020) and obtained from Qiagen (Supplementary Table S7). SiRNA forward transfections with Lipofectamine RNAiMAX (Invitrogen) were performed on two consecutive days. The concentration of siRNAs in transfections was 20 nM each. Cells were treated with drugs at 96 h after the first transfection.

### Generation of *RAD54L*/*RAD51AP1* and *RAD54L*/*RAD54B* DKO HeLa Cells

*RAD51AP1* knockout (KO) and *RAD54L* KO HeLa cells, that we described previously (Liang et al., 2019; Maranon et al., 2020), were used to generate *RAD54L/RAD51AP1* and *RAD54L*/*RAD54B* DKO cells. Briefly, a combination of two *RAD54L* or *RAD54B* CRISPR/Cas9-nic (D10A) KO plasmids each containing one of two different sgRNAs (i.e., sgRNA (54)-A and sgRNA (54)-B; sgRNA (54B)-A and sgRNA (54B)-B; Supplementary Table S5) was purchased from Santa Cruz Biotechnology (sc-401750-NIC for *RAD54L*; sc-403794-NIC-2 for *RAD54B*) and used to transfect single KO cells as described (Maranon et al., 2020). Disruption of *RAD54L* and *RAD54B* was validated by sequence analysis after genomic DNA was isolated from a selection of edited and non-edited clonal isolates using DNeasy Blood & Tissue Kit (Qiagen). *RAD54L* and *RAD54B* genomic DNA sequences were amplified by PCR using primer pairs flanking the sgRNA target sites (Supplementary Table S6). PCR products were gel purified, cloned into pCR4-TOPO (Invitrogen) and transformed into TOP10 competent *E. coli*. Plasmid DNA was prepared using ZR Plasmid Miniprep-Classic Kit (Zymo Research) and submitted for sequencing. For each KO cell line, 15–20 individually cloned amplicons were analyzed by Sanger sequencing (Supplementary Table S1).

### Generation of *RAD51AP1* and *RAD54L* Single KO and DKO Hs578T Cells

The *RAD54L* CRISPR/Cas9-nic (D10A) KO plasmids described above (Supplementary Table S5) were used to generate Hs578T *RAD54L* KO cells. Hs578T cells and *RAD54L* KO cells then were transfected with a cocktail of three different CRISPR/Cas-9 knockout plasmids (Santa Cruz Biotechnology; sc-408187) each encoding Cas9 nuclease and one of three different *RAD51AP1*-specific gRNAs targeting exons 2, 3 or 5/6 (Supplementary Table S5). Clonal isolates were expanded and disruption of *RAD54L* and *RAD51AP1* was validated by sequence analysis, as described above. For each KO cell line, 15–20 individually cloned amplicons were analyzed by Sanger sequencing (Supplementary Table S1).

### Generation of RAD54L-Expressing *RAD51AP1* and/or *RAD54L* KO HeLa Cells

The plasmid containing the C-terminally HA-tagged full-length human RAD54L cDNA has been described (Maranon et al., 2020). A *Kpn*I to *Not*I digest was performed to clone RAD54L-HA into pENTR1A (Invitrogen), followed by transfer into pLentiCMV/TO DEST#2 (Campeau et al., 2009) using Gateway LR Clonase II (Invitrogen) for the production of lentiviral particles in HEK293FT cells (Invitrogen), as described (Campeau et al., 2009). Lentivirus was used to transduce *RAD51AP1* KO, *RAD54L* KO, and *RAD54L*/*RAD51AP1* DKO cells in 6 μg/ml polybrene, as described (Campeau et al., 2009).

### Clonogenic Cell Survival Assays and Western Blot Analysis

Clonogenic cell survival assays after mitomycin C (MMC; Sigma) were performed, as described (Maranon et al., 2020). To assess cellular sensitivity to olaparib (AZD2281; Selleck Chemicals), cells were chronically exposed to 0.5–4 μM olaparib in regular growth medium for 12–14 days, as described (Spies et al., 2016). Cells were fixed and stained with crystal violet to determine the fraction of cells surviving.

Western blot analyses were performed according to our standard protocols (Wiese et al., 2006). The following primary antibodies were used: α-RAD51AP1 (Dray et al., 2010; 1:6,000), α-RAD54L (F-11; sc-374598; Santa Cruz Biotechnology; 1:1,000); α-RAD51 (Ab-1; EMD Millipore; 1:3,000), α-PARP1 (ab6079; Abcam; 1:2,000), α-β-Actin (ab8226; Abcam; 1:1,000), α-Tubulin (DM1A; Santa Cruz Biotechnology; 1:3,000), α-HA.11 (MMS-101R; BioLegend; 1:1,000), α-Histone H3 (ab1791; Abcam; 1:10,000) and α-RAD54B (Wesoly et al., 2006; 1:1,000). HRP-conjugated goat anti-rabbit or goat anti-mouse IgG (Jackson ImmunoResearch; 1:10,000) were used as secondaries. Western blot signals were acquired using a Chemidoc XRS+ gel imaging system and ImageLab software version 5.2.1 (BioRad).

### Cell Cycle Analysis and Flow Cytometry

Cell cycle analysis and flow cytometry were performed as described (Maranon et al., 2020), except that exponentially growing cells were treated with 0.5 μM MMC for 2 h, washed twice with warm PBS and incubated in fresh growth medium for the times indicated prior to pulse-labeling with 10 μM EdU.

### Metaphase Spreads

For the assessment of chromosomal aberrations, 2 × 10^5^ cells were seeded in 6-well tissue culture plates and incubated at 37°C for 24 h before exposure to 4 mM hydroxyurea (HU; Sigma) in regular growth medium for 5 h, as described (Schlacher et al., 2011). After HU treatment, cells were washed in warm PBS and incubated in medium containing 0.1 μg/ml colcemid (SERVA) for 24 h. Cells were detached and allowed to swell in 0.075 M KCl at 37°C for 30 min and fixed in methanol:acetic acid (3:1), as described (Parplys et al., 2015). Cells were dropped onto wet slides, air dried and stained in 3% Giemsa in Sorensen buffer (0.2 M Na_2_HPO_4_/NaH_2_PO4, pH 7.3) at room temperature for 10 min. Images were acquired using Zeiss Axio-Imager.Z2 microscope equipped with Zen Blue software (Carl Zeiss Microscopy) using a 63× oil objective. One hundred metaphases were assessed per sample.

### DNA Fiber Assay

DNA replication progression was assessed by the single-molecule DNA fiber assay and essentially as described previously (Schlacher et al., 2011; Parplys et al., 2015; Taglialatela et al., 2017). Briefly, exponentially growing cells were pulse-labelled in regular growth medium containing 25 μM CldU for 20 min, followed by a 5-hour incubation in regular growth medium with 4 mM HU, after which the cells were pulse-labelled in regular growth medium containing 250 μM IdU for 20 min. Cells were detached from the cell culture dish by scraping in ice-cold PBS, adjusted to 4 × 10^5^ cell/ml and processed for fiber spreading as described (Parplys et al., 2015). In a modified version of this assay, cells were exposed for 24 h in 10 μM olaparib, followed by two consecutive rounds of 20 min each in CldU first and then in IdU (Maya-Mendoza et al., 2018). Images were acquired using Zeiss Axio-Imager.Z2 microscope equipped with Zen Blue software (Carl Zeiss Microscopy) using a 63× oil objective. Per sample and condition 200 fiber tracts were measured using ImageJ software (https://imagej.net).

### Co-Immunoprecipitations

The peGFP-RAD51AP1 expression vector is based on peGFP-C1 (Clontech) and has been described previously (Modesti et al., 2007). *RAD51AP1* single or *RAD54L*/*RAD51AP1* double KO cells were transfected with peGFP-C1 or peGFP-RAD51AP1 and Lipofectamine2000 (Invitrogen). Twenty-four hours after transfection, cells were subjected to a medium change, treated with 0.5 µM MMC for 2 h or 10 µM olaparib for 24 h. Cells were washed twice with warm PBS, fresh medium was added, and cells were incubated for the times indicated. Cells were lysed in chilled lysis buffer containing 50 mM Tris-HCl, pH 7.5, 300 mM NaCl, and 0.5% NP-40, supplemented with EDTA-free protease inhibitor cocktail (Roche) and HALT phosphatase inhibitors (Thermo Fisher Scientific). For 1.5 × 10^6^ cells, 25 μl of GFP-Trap^®^ dynabeads (ChromoTek) were used to trap the ectopic proteins. Protein lysates were diluted to 50 mM Tris-HCl, pH 7.5, 150 mM NaCl, 0.1% NP-40, and 0.1 unit DNase I (Gold Biotechnology) per µg protein, and mixed with the equilibrated beads at 4°C for 1 h with gentle rotation. The GFP-Trap^®^ dynabeads were washed three times with 500 µl binding buffer, bound protein complexes were eluted in 40 µl 2× LDS buffer (Thermo Fisher Scientific) and fractionated on 7% NuPAGE Tris-Acetate gels (Thermo Fisher Scientific) and for Western blot analysis.

### Purification of Recombinant Proteins and FLAG Pull-Downs

Expression of (His)_6_-RAD51AP1-FLAG in *E. coli* and its purification were carried out as described previously (Maranon et al., 2020). RAD54B was expressed in High Five insect cells transduced with a RAD54B baculovirus and purified as described (Sehorn et al., 2004).

FLAG pull-downs were performed essentially as described (Maranon et al., 2020). Briefly, anti-FLAG M2 affinity resin was equilibrated in binding buffer (50 mM Tris-HCl, pH7.5, 150 mM NaCl, 0.1% Triton X-100, and 100 μg/ml BSA). (His)_6_-RAD51AP1-FLAG (100 nM) or no protein were incubated with the equilibrated resin at 4°C for 1 h. Unbound protein was removed by centrifugation at 3,000 rpm for 3 min RAD54B (100 nM) was added to the washed resin in 100 µl binding buffer and incubated at 4°C for 1 h with gentle agitation in the presence of DNase I (1 U/µg protein). Supernatant was removed and RAD54B (100 nM) was added in a final volume of 100 µl and further incubated for 1 h at 4°C. The resin was washed three times in 200 µl binding buffer each, and bound protein was eluted in binding buffer containing 150 ng/μl 3× FLAG peptide (Sigma). Eluted protein was fractionated by 10% SDS-PAGE, transferred onto a PVDF membrane and detected by Western blot analysis.

### Statistics and Reproducibility

GraphPad Prism 9 software was used to perform statistical analyses on data obtained from two to five independent experiments, as indicated. To assess statistical significance two-way or one-way ANOVA tests were performed. *p* ≤ 0.05 was considered significant.

## Data Availability

The original contributions presented in the study are included in the article/Supplementary Material, further inquiries can be directed to the corresponding author.
