# Supplementary material for: RAD51AP1 and RAD54L Can Underpin Two Distinct RAD51-Dependent Routes of DNA Damage Repair via Homologous Recombination
Source: Front Cell Dev Biol. 2022 May 16;10:866601. doi: 10.3389/fcell.2022.866601 (PMC9149245; doi:10.3389/fcell.2022.866601)
Supplement: Supplementary file 1 [file Presentation1.pdf]

# *Supplementary Material*

## 1 Supplementary Materials and Methods

### 1.1 Cell Fractionation and Cell Growth Assay

Cell fractionation was carried out using the Subcellular Protein Fractionation Kit (Thermo Fisher Scientific) and as described by the manufacturer. To determine the rates of cell growth, cells were seeded in 6-well tissue culture plates at a density of $1.2\times10^4$ cells/well. Cells were counted after trypsinization for eight consecutive days. Cell numbers were plotted using nonlinear regression, and population doubling times (PDL) were calculated using the following equation:

$$\mathrm{PDL} = 3.32 \times \log\left(\frac{\text{total viable cells at harvest}}{\text{total viable cells at seed}}\right)$$

### 1.2 Recombination Assay

U2OS-DRGFP (*i.e.* DR-U2OS) cells have been described elsewhere (Nakanishi et al., 2005; Xia et al., 2006). The gene conversion assay using U2OS-DRGFP cells was performed as previously described (Liang et al., 2016; Liang et al., 2020; Parplys et al., 2015). Briefly, $1.5\times10^5$ cells were seeded in 6-well tissue culture plates 24 hours prior to transfection in antibiotic free media. I-*Sce*I was expressed transiently in U2OS-DRGFP cells from the pCβASCE expression vector (Richardson et al., 1998) at 0.8 µg/150,000 cells and co-transfected with siRNA using Lipofectamine2000 (Invitrogen). Transfected cells were kept in regular growth medium for 72 hours, after which time they were analyzed by flow cytometry to measure the percentage of viable cells expressing GFP, as previously described (Parplys et al., 2015). To assess the fraction of GFP-positive cells, FlowJo version 10.7 (BD Biosciences) was used.

### 1.3 Indirect Immunostaining, Microscopy, and Image Analysis

Indirect immunostaining was performed as described (Maranon et al., 2020). The primary antibody used to detect RAD54 foci was α-RAD54 (F-11; sc-374598; Santa Cruz Biotechnology; 1:1,000). A Zeiss Axio-Imager.Z2 microscope equipped with Zen Blue software (Carl Zeiss Microscopy) was used to take images using a 63× oil objective. In each channel, 18 Z-stacks were obtained as 0.2 µm slices. Images were processed in Fiji (https://imagej.net/Fiji), separating the channels and producing maximum projection files.

### 1.4 Co-Immunoprecipitations

*RAD51AP1* KO cells stably expressing FLAG-tagged RAD51AP1 were used. *RAD51AP1* was introduced into *RAD51AP1* KO cells by transduction with lentivirus, as described previously (Campeau et al., 2009). Protein lysate from HeLa with endogenous RAD51AP1 (control) and from *RAD51AP1* KO cells expressing FLAG-RAD51AP1 were prepared in chilled buffer containing 50 mM Tris-HCl, pH 7.5, 300 mM NaCl, and 0.5% NP-40 supplemented with EDTA-free protease inhibitor cocktail (Roche) and HALT phosphatase inhibitors (ThermoScientific). Protein lysates were adjusted to 150 mM NaCl, 0.1% NP-40, and 0.1 unit/µg protein DNase I (GoldBio). Twenty-five µl ANTI-FLAG ® M2- resin (Sigma) was equilibrated with binding buffer (50 mM Tris-HCl, pH 7.5,

150 mM NaCl and 0.1% NP-40) before protein lysates containing 2 mg total protein were added and incubated for at 4°C for 1 hour with gentle rotation. Protein complexes bound to anti-FLAG resin were washed three times with 500 µl binding buffer, and bound protein complexes were eluted with 150 ng/µl 3× FLAG peptide (Sigma) in binding buffer. Eluted protein was fractionated on 7.5% NuPAGE Tris-Acetate gels, transferred onto polyvinylidene fluoride membrane (Millipore), and detected by Western blot analysis.

### 1.5 Comet Assay

The Comet assay (Trevigen) was performed according to the manufacturer's instructions. Briefly, $5\times10^4$ cells were seeded in 60-mm plates. Cells were incubated at 37°C for 48 h and then treated with 10 µM olaparib or DMSO for 24 h. Cells were harvested in cold PBS, counted and combined with molten low melting (LM)agarose at 1:10 to $1\times10^5$ cells/ml. Fifty µl of the suspension were transferred on to comet slides and incubated in the dark at 4°C for 30 minutes. Slides were incubated in cold lysis buffer at 4°C overnight. Excess lysis buffer was drained and electrophoresis was performed in cold neutral comet electrophoresis buffer (10 mM Tris base, 250 mM sodium acetate) at 25 V and 4°C for 30 min. The slides were immersed in DNA precipitation solution (Trevigen) for 30 min, followed by incubation in 70% ethanol for 30 min. Slides were the dried at 37°C for 30 min and stained with 0.3× SYBR Gold (Thermo Fisher). Images were acquired on a Zeiss Axio-Imager.Z2 microscope equipped with Zen Blue software (Carl Zeiss Microscopy) using a 20× objective, and 100 comets were measured per condition. The length comet tails were measured using ImageJ software (https://imagej.net).

## 2 Supplementary References

Adey, A., J.N. Burton, J.O. Kitzman, J.B. Hiatt, A.P. Lewis, B.K. Martin, R. Qiu, C. Lee, and J. Shendure. 2013. The haplotype-resolved genome and epigenome of the aneuploid HeLa cancer cell line. *Nature*. 500:207-211.

Campeau, E., V.E. Ruhl, F. Rodier, C.L. Smith, B.L. Rahmberg, J.O. Fuss, J. Campisi, P. Yaswen, P.K. Cooper, and P.D. Kaufman. 2009. A versatile viral system for expression and depletion of proteins in mammalian cells. *PLoS One*. 4:e6529.

Hackett, A.J., H.S. Smith, E.L. Springer, R.B. Owens, W.A. Nelson-Rees, J.L. Riggs, and M.B. Gardner. 1977. Two syngeneic cell lines from human breast tissue: the aneuploid mammary epithelial (Hs578T) and the diploid myoepithelial (Hs578Bst) cell lines. *Journal of the National Cancer Institute*. 58:1795-1806.

Liang, F., S. Longerich, A.S. Miller, C. Tang, O. Buzovetsky, Y. Xiong, D.G. Maranon, C. Wiese, G.M. Kupfer, and P. Sung. 2016. Promotion of RAD51-Mediated Homologous DNA Pairing by the RAD51AP1-UAF1 Complex. *Cell reports*. 15:2118-2126.

Liang, F., A.S. Miller, C. Tang, D. Maranon, E.A. Williamson, R. Hromas, C. Wiese, W. Zhao, P. Sung, and G.M. Kupfer. 2020. The DNA-binding activity of USP1-associated factor 1 is required for efficient RAD51-mediated homologous DNA pairing and homology-directed DNA repair. *J Biol Chem*. 295:8186-8194.

Maranon, D.G., N. Sharma, Y. Huang, P. Selemenakis, M. Wang, N. Altina, W. Zhao, and C. Wiese. 2020. NUCKS1 promotes RAD54 activity in homologous recombination DNA repair. *J Cell Biol*. 219.

Nakanishi, K., Y.G. Yang, A.J. Pierce, T. Taniguchi, M. Digweed, A.D. D'Andrea, Z.Q. Wang, and M. Jasin. 2005. Human Fanconi anemia monoubiquitination pathway promotes homologous DNA repair. *Proc Natl Acad Sci U S A*. 102:1110-1115.

Parplys, A.C., W. Zhao, N. Sharma, T. Groesser, F. Liang, D.G. Maranon, S.G. Leung, K. Grundt, E. Dray, R. Idate, A.C. Ostvold, D. Schild, P. Sung, and C. Wiese. 2015. NUCKS1 is a novel RAD51AP1 paralog important for homologous recombination and genome stability. *Nucleic Acids Res*. 43:9817-9834.

Richardson, C., M.E. Moynahan, and M. Jasin. 1998. Double-strand break repair by interchromosomal recombination: suppression of chromosomal translocations. *Genes & development*. 12:3831-3842.

Xia, B., Q. Sheng, K. Nakanishi, A. Ohashi, J. Wu, N. Christ, X. Liu, M. Jasin, F.J. Couch, and D.M. Livingston. 2006. Control of BRCA2 cellular and clinical functions by a nuclear partner, PALB2. *Molecular cell*. 22:719-729.

# 3 Supplementary Figures and Tables

## 3.1 Supplementary Figures

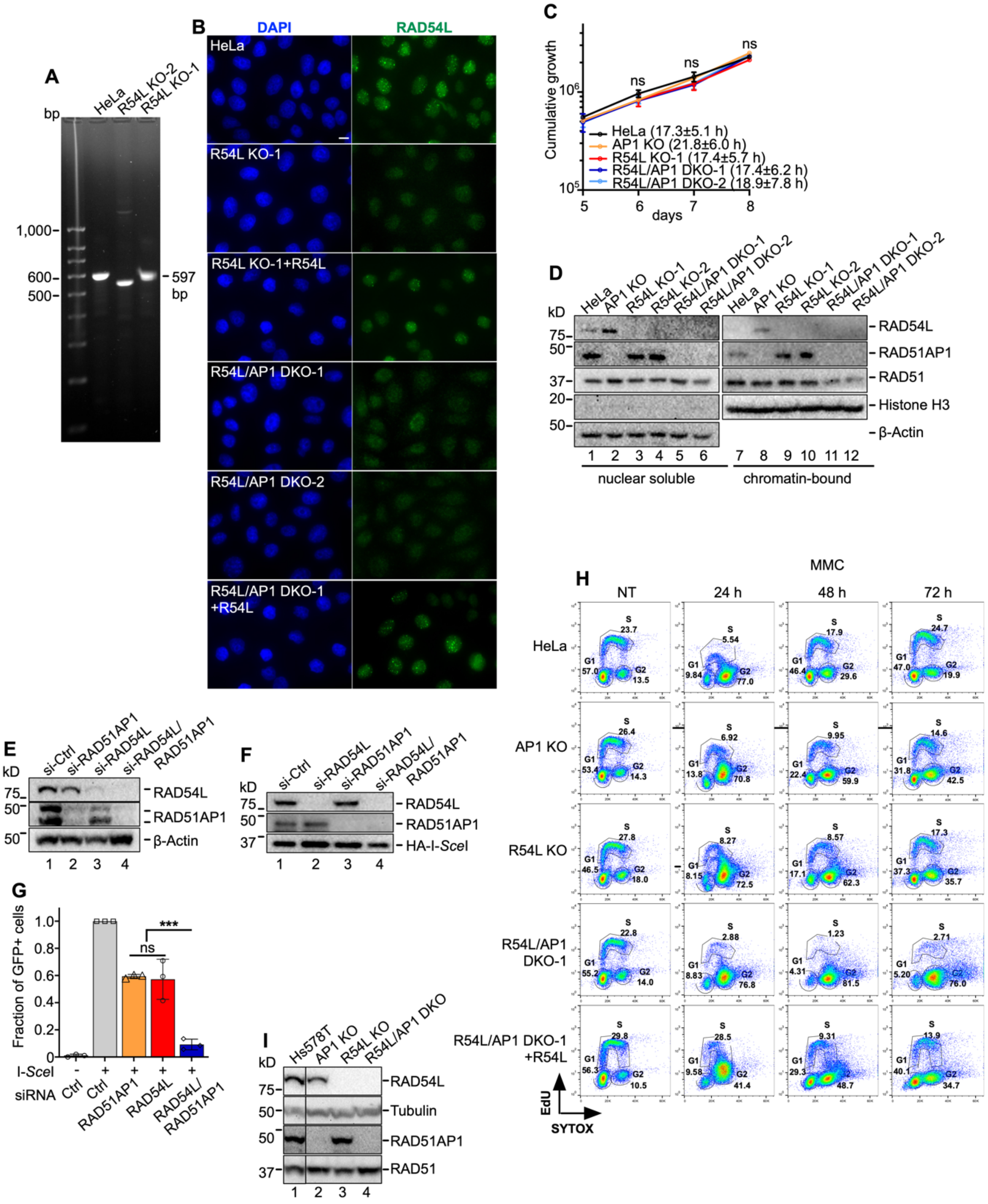


**Supplementary Figure 1.** Generation, verification and cell growth and cell cycle analysis of single and double KO cell lines derived from HeLa cells, complementation of the *RAD54L* single and *RAD54L/RAD51AP1* double KOs by ectopic RAD54L-HA, verification of protein knockdown in A549 and U2OS cells, and generation of single and double KOs from Hs578T cells. **(A)** Representative agarose gel of PCR products for *RAD54L* obtained from genomic DNA of HeLa, *RAD54L* KO-1 and

*RAD54L* KO-2 cells using primers P1 and P2 (**Supplementary Table 6**). **(B)** Representative micrographs of RAD54L foci (green) in HeLa, *RAD54L* KO-1, *RAD54L*/*RAD51AP1* double KO (KO-1 and KO-2) cells, and in *RAD54L* KO-1 and *RAD54L*/*RAD51AP1* KO-1 cells stably expressing RAD54L-HA (here: +R54L); scale bar: 10 µm. **(C)** Growth curves under unperturbed conditions of HeLa and KO cell lines. Data points are the means from three experiments ±SD. ns, non-significant; two-way ANOVA test followed by Tukey's multiple comparisons test. **(D)** Western blots of soluble and chromatin-bound nuclear extracts of HeLa cells and single and double KO cell lines. The signals for β-Actin and histone H3 serve as a loading and fractionation control, respectively. **(E)** Western blots of whole cell protein lysates to show the extent of RAD54L/RAD51AP1 protein knockdown in A549 cells. Loading control: β-Actin. **(F)** Western blots of nuclear extracts to show the extent of RAD54L/RAD51AP1 protein knockdown and I-*Sce*I expression in U2OS-DRGFP cells. **(G)** Average percentage of GFP-positive cells normalized to non-targeting control siRNA (Ctrl) after RAD51AP1 and/or RAD54L knockdown in U2OS-DRGFP cells. Bars are the means from three independent experiments ±SD. Symbols are data points from individual experiments. ***, $p<0.001$; ns, non-significant; one-way ANOVA test followed by Tukey's multiple comparisons test. **(H)** Representative results from flow cytometry showing two-color fluorescence of cell cycle profiles of the cells used in **Figures 1D** and **2B**. Y-axis: EdU, X-axis: SYTOX. **(I)** Western blots of whole cell protein lysates of Hs578T cells and generated KO cell lines. Loading control: Tubulin.

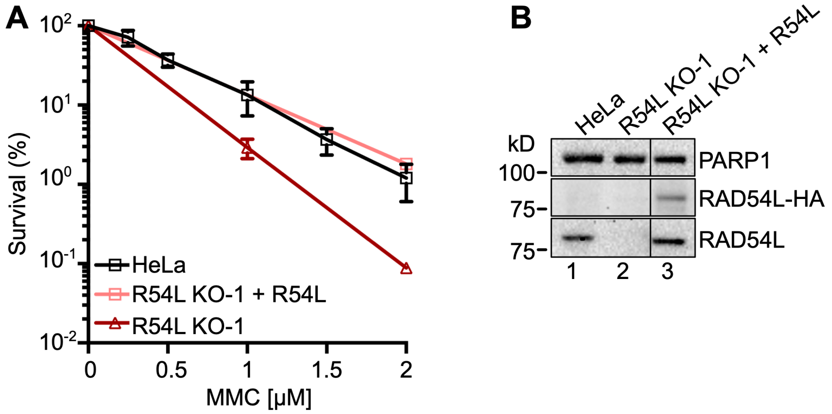


**Supplementary Figure 2.** Stable expression of ectopic RAD54L-HA in *RAD54L* KO-1 cells rescues MMC cytotoxicity. **(A)** Results from MMC clonogenic cell survival assays of HeLa cells and *RAD54L* KO (R54L KO-1) cells with and without ectopic RAD54L-HA (here: +R54L). Data points for R54L KO-1+R54L are the means from three technical replicates. Data points for R54L KO-1 and HeLa cells are the means from three independent experiments ±SD. **(B)** Western blots of whole cell protein extracts to show stably expressed ectopic RAD54L-HA in R54L KO-1 cells (lane 3), and comparable levels of endogenous and ectopic RAD54L in HeLa and R54L KO-1+R54L cells (compare lanes 1 and 3, respectively). Loading control: PARP1.

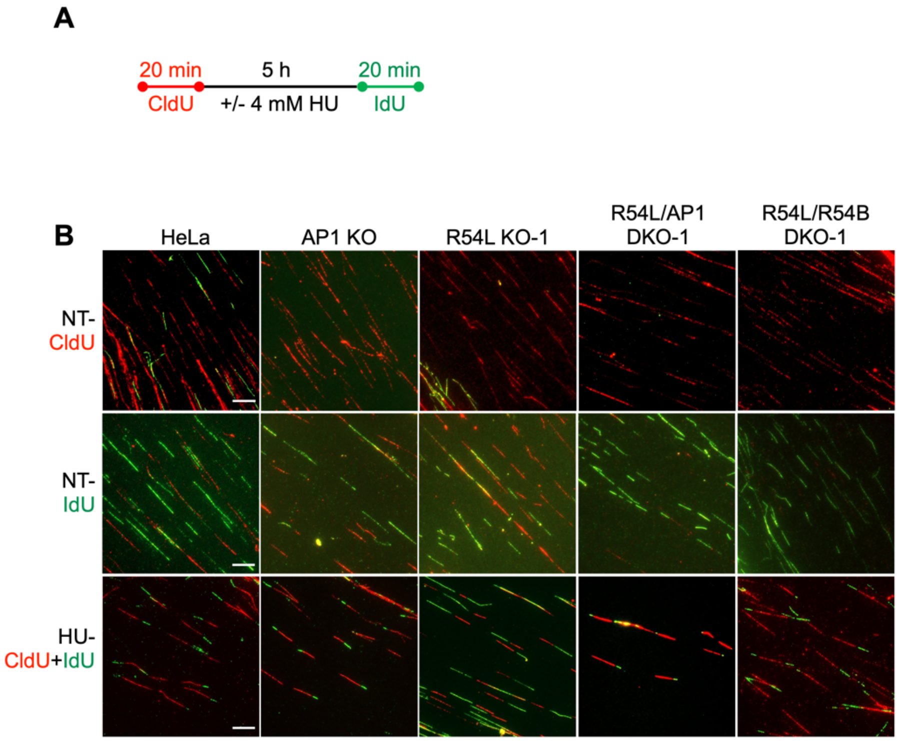


**Supplementary Figure 3**. Replication restart after HU is significantly impaired in *RAD54L*/*RAD51AP1* DKO cells. **(A)** Schematic of experimental protocol of the DNA fiber assay with and without HU. **(B)** Representative micrographs of DNA fibers from HeLa, single, and double KO cells. NT: non-treated. Scale bars:10 μm.

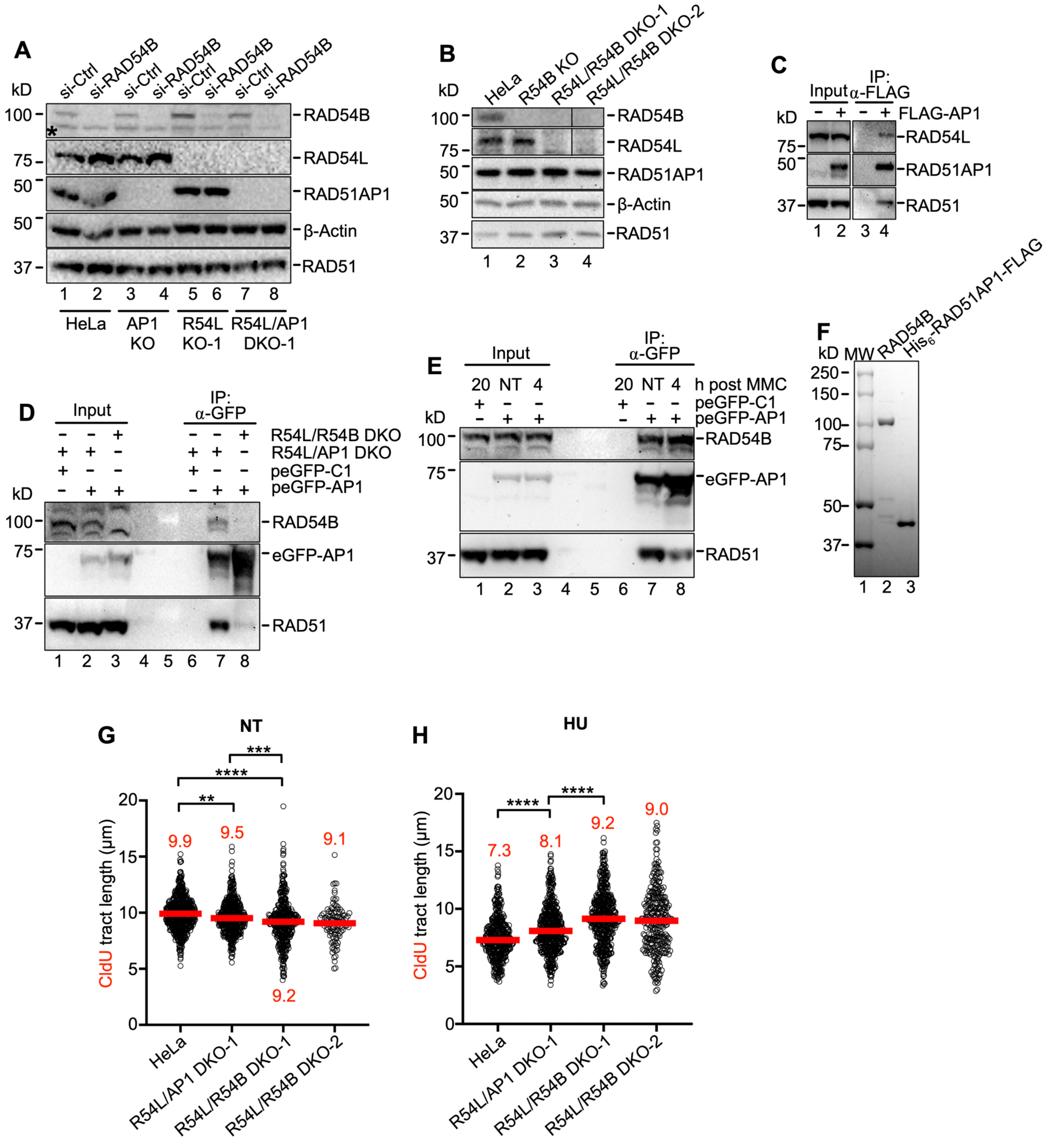


**Supplementary Figure 4**. Verification of RAD54B knockdown and *RAD54B* loss, co-immunoprecipitation of endogenous RAD54L or RAD54B with ectopic RAD51AP1, and CldU tracts after HU are not further degraded in DKO compared to HeLa cells. **(A)** Western blots of whole cell protein extracts of HeLa (lanes 1 and 2), *RAD51AP1* single KO (lanes 3 and 4), *RAD54L* single KO-1 (lanes 5 and 6) and *RAD54L*/*RAD51AP1* (lanes 7 and 8) double KO-1 cells. Loading control: β-Actin. Asterisk: non-specific band. **(B)** Western blots of whole cell protein extracts to show loss of expression of both RAD54B and RAD54L in two independently isolated *RAD54L*/*RAD54B* DKO cell lines (KO-1 and KO-2; lanes 3 and 4). Loading control: β-Actin. **(C)** Western blots to show that endogenous

RAD54L co-precipitates in anti-FLAG co-immunoprecipitations (IPs) of stably expressed ectopic FLAG-RAD51AP1 in *RAD51AP1* KO cells (lane 4). The signal for RAD51 serves as a positive control. **(D)** Western blots to show that endogenous RAD54B co-precipitates in anti-GFP IPs of ectopically expressed GFP-RAD51AP1 in *RAD54L*/*RAD51AP1* DKO cells (lane 7), but not in anti-GFP IPs of ectopically expressed GFP-RAD51AP1 in *RAD54L*/*RAD54B* DKO cells (lane 8). The signal for RAD51 serves as a positive control. Note: Diminished RAD51 is detected in anti-GFP IPs of GFP-RAD51AP1 expressed in *RAD54L*/*RAD54B* DKO cells (lane 8) likely due to endogenous RAD51AP1 interfering with complex formation between GFP-RAD51AP1 and RAD54B. **(E)** Western blots to show that endogenous RAD54B co-precipitates in anti-GFP IPs of ectopically expressed GFP-RAD51AP1 in *RAD54L*/*RAD51AP1* DKO cells, constitutively and at 4 h post release from a 2-h incubation of cells in 0.5 μM MMC (lanes 7 and 8, respectively). (**F)** SDS-PAGE of purified RAD54B and $(His)_6$-RAD51AP1-FLAG (1 μg each). (**G**, **H)** Median CldU (red) tract lengths in cells without (NT) or with HU treatment. Data points are from 100-150 fibers of three independent experiments for HeLa, *RAD54L*/*RAD51AP1* DKO-1 and *RAD54L*/*RAD54B* DKO-1 cells, with medians indicated (red). ****, $p<0.0001$; ***, $p<0.001$; **, $p<0.01$; Kruskal-Wallis test followed by Dunn's multiple comparisons test. Data points for RAD54L/RAD54B DKO-2 cells are from 1 experiment with 100 fibers analyzed.

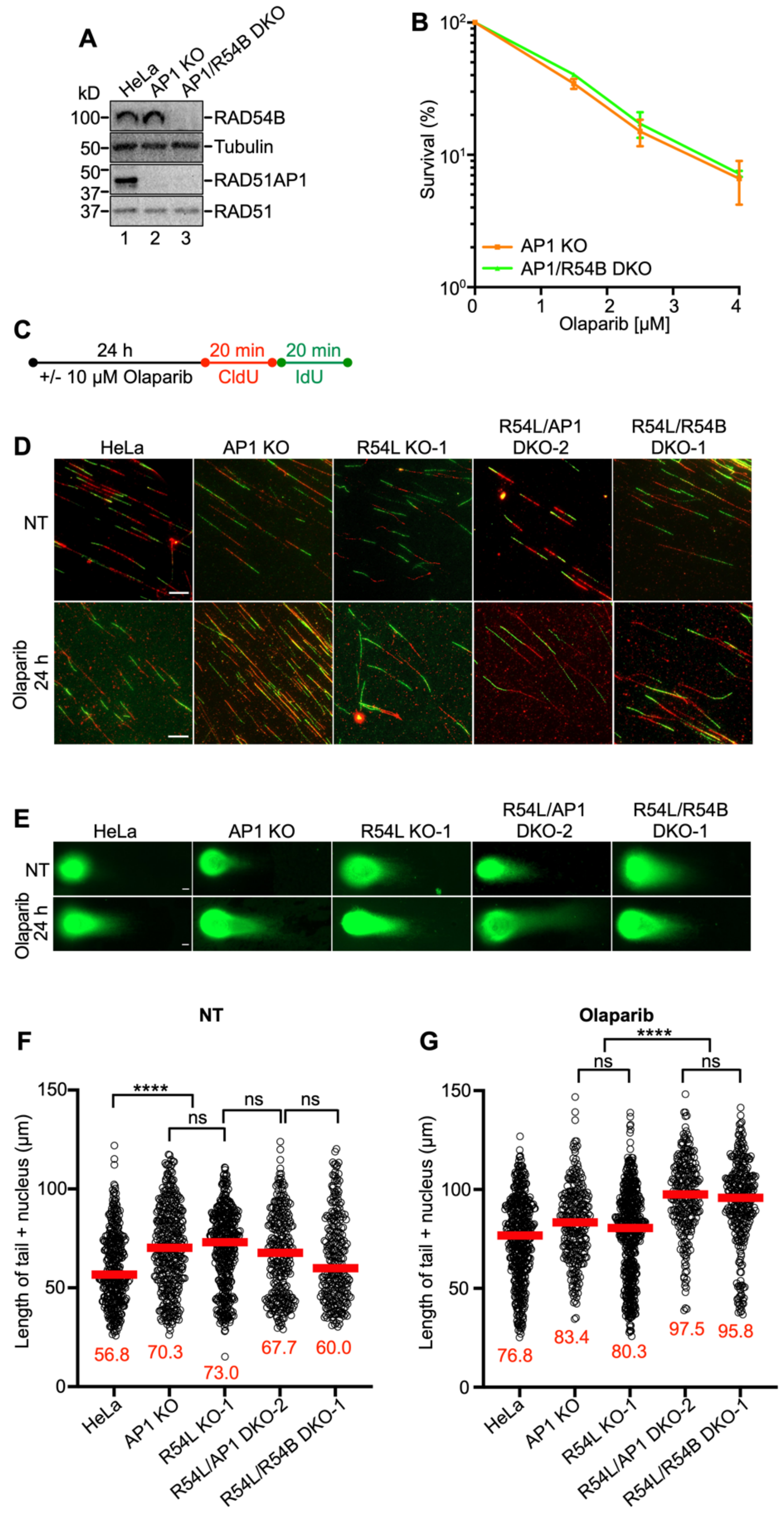


**Supplementary Figure 5.** Impact of olaparib treatment on *RAD51AP1*/*RAD54B* DKO cell survival, and on replication fork progression and double-strand break formation. **(A)** Western blots of whole cell protein extracts of HeLa, *RAD51AP1* single KO (lane 2) and *RAD51AP1*/*RAD54B* DKO cells (lane 3).

Loading control: Tubulin. **(B)** Results from olaparib clonogenic cell survival assays of *RAD51AP1* (AP1) KO and *RAD51AP1*/*RAD54B* (AP1/R54B) DKO cells. Data points are the means from three independent experiments ±SD. **(C)** Schematic of the protocol of the DNA fiber assay used for the micrographs shown in (E). **(D)** Representative micrographs of DNA fibers in unperturbed cells (NT) and after a 24-h incubation in 10 µM olaparib. Scale bars: 10 µm. **(E)** Representative micrographs of nuclei with eluted DNA after neutral comet assay of unperturbed cells (NT) and after a 24-h incubation in 10 µM olaparib. Scale bars: 10 µm. **(F, G)** Quantification of comets of unperturbed cells (NT) and after a 24-h incubation in 10 µM olaparib. Data points are from 120-230 nuclei of 2-3 independent experiments each (**Supplementary Table 4**). ****, $p < 0.0001$; ns, non-significant; Kruskal-Wallis test followed by Dunn's multiple comparisons test.

## 3.2 Supplementary Tables

**Supplementary Table 1.** Sequencing results for genotyping of *RAD51AP1*, *RAD54L* and *RAD54B* disrupted cells.

| Gene, estimated copy number*, chromosome | Cell line | Genomic sequence (gRNA-PAM)# | Indel (nt) |
|---|---|---|---|
| *RAD51AP1* 3 Chr. 12 | AP1 KO (HeLa) | AATGAATAAGTCTCCTCATATCTCTAATTGCAGTGTAGCCAGTGATTATTTAGGTAAGTTTTTTATATTAATAATT | WT$_{E6}$ |
| | | AATGAATAAGTCTCCTCATATCTCTAATTGCAGTGTAGCCAGTGATTAaTTTAGGTAAGTTTTTTATATTAATAAT T | +1 |
| | | AATGAATAAGTCTCCTCATATCTCTAATTGCAGTGTAGCCAGTGA----TTAGGTAAGTTTTTTATATTAATAATT | -4 |
| | | AATGAATAAGTCTCCTCATATCTCTAATTGCAGTGTAGCCAG-----------GTAAGTTTTTTATATTAATAATT | -11 |
| *RAD54L* 3 Chr. 1 | R54L KO-1 (HeLa) | GCCTTCCAGCCTGGTGAAGAACTGGTACAATGAGGTTGGGAAATGGCTCGGAGGGAGGATCCAACCTCTGGCCATC | WT |
| | | GCCTTCCAG-------------------------GTTGGGAAATGGCTCGGAGGGAGGATCCAACCTCTGGCCATC | -25 |
| | | GCCTTCCAGCC-----------------AATGAGGTTGGGAAATGGCTCGGAGGGAGGATCCAACCTCTGGCCATC | -17 |
| | | GCCTTCCAGCCTGGTGAc-------------------------------------AGGATCCAACCTCTGGCCATC | -37 |
| *RAD54L* 3 Chr. 1 | R54L KO-2 (HeLa) | GCCTTCCAGCCTGGTGAAGAACTGGTACAATGAGGTTGGGAAATGGCTCGGAGGGAGGATCCAACCTCTGGCCATC | WT |
| | | GCCTTCC-----------------------------------------------------CCAACCTCTGGCCATC | -53 |
| | | | |
| | | | |
| *RAD54L* 3 Chr. 1 | R54L/ AP1 KO-1 (HeLa) | GCCTTCCAGCCTGGTGAAGAACTGGTACAATGAGGTTGGGAAATGGCTCGGAGGGAGGATCCAACCTCTGGCCATC | WT |
| | | GCCTTCCAGCCTGGTGAAGAACTGGTACAATGAGGTTGGGAAATGGgTac----aatGATCCAACCTCTGGCCATC | -4 |
| | | GCCTTCCAGCCTGGT-----------------------------------------GGATCCAACCTCTGGCCATC | -41 |
| | | GCCTTC---------------------------//----------------------------------------- | -251 |
| *RAD54L* 3 Chr. 1 | R54L/ AP1 KO-2 (HeLa) | GCCTTCCAGCCTGGTGAAGAACTGGTACAATGAGGTTGGGAAATGGCTCGGAGGGAGGATCCAACCTCTGGCCATC | WT |
| | | GCCTTCCAGCCTGGT-----------------------------------------GGATCCAACCTCTGGCCATC | -41 |
| | | GCCTTCCAGttgtttc------------------//---------------------------------------- | -249 |
| | | GCCTTCCAGCCTGGTGAAGAACTGGTACAATGAGGTTGGGAAATG----GGtacaAtGATCCAACCTCTGGCCATC | -4 |
| *RAD54B* 4 Chr. 8 | R54L/ R54B KO-1 (HeLa) | GTTATGCCACGACCAGATAAGAATCACCAGTGGGTATTCAATAAGAACTGTTTCCCTCTTGTGGATGTAGTGATTG | WT |
| | | GTTATGCCACGACCAGATAAGAATCACCAGTGGGTATTCAATAAGAACTGTTTCCCTCcaGTGGgtatactggtat tcaataagaactgtttccctccagtgggtattcaataagaactgttGATTG | +51 |
| | | GTTATGCCACGACCAGATAAGAATCACCAGTGGGTATTCAATAAGAACT-----------GTGGATGTAGTGATTG | -11 |
| | | GTTATGCCACGACCAGATAAGAATacC-------------------------cCaCTCTTGTGGATGTAGTGATTG | -25 |
| | | GTTATGCCACGACCAGATAAGAATCACCAGTGGGTA----------------------//---------------- | -375 |
| *RAD54B* 4 Chr. 8 | R54L/ R54B KO-2 (HeLa) | GTTATGCCACGACCAGATAAGAATCACCAGTGGGTATTCAATAAGAACTGTTTCCCTCTTGTGGATGTAGTGATTG | WT |
| | | GTTATGCCACGACCAGATAAGAATCACCAGTtctTATT----------------------GTGGATGTAGTGATTG | -22 |
| | | GTTATGCCACGACCAGATAAGAATCACCAGTG-------------------------------GATGTAGTGATTG | -31 |
| | | GTTATGCCACGACCAGATAAGAATCACCAGTGGGTA--------------------------------------TG | -38 |
| | | | |

| | | | |
|---|---|---|---|
| *RAD54B*<br>4<br>Chr. 8 | AP1/<br>R54B<br>KO<br>(HeLa) | GTTATGCCACGACCAGATAAGAATCACCAGTGGGTATTCAATAAGAACTGTTTCCCTCTTGTGGATGTAGTGATTG | WT |
| | | GTTATGCCACGACCAGATAAGAATCACCAGTGGGTATTCAATAAGAACTGTTTCCCTCagtgggtattcaataaga<br>actgtttccctcagtgggtattcaataagaactgtttccctcTGTGGATGTAGTGATTG | +59 |
| | | GTTATGCCACGACCAGATAAGAATCACCAGTGGGTATTCAAtaagacagataagaatcaccagtgggtattctcac<br>cagtgggtattcaataagacagataagaatcaccagtgggtattcaaTAAGAACTGTTTCCCTCTTGTGGATGTAG | +82 |
| | | GTTATGCCACGACCAGATAA----tAggAGTGGGTATTCAATAAGAACTGTTTCCCTCTTGTGGATGTAGTGATTG | -4 |
| | | | |
| *RAD51AP1*<br>3<br>Chr. 12 | AP1<br>KO-1<br>(Hs578T) | GGAGTTAAAACAAGATAAACCAAAACCTAACTTGAACAATCTCCGGAAAGAAGAAATCCCAGTACAAGAGAAAACC | WT$_{E3}$ |
| | | GGAGTTAAAACAAGATAAACCAAAACCTAACTTGAACAATC--CGGAAAGAAGAAATCCCAGTACAAGAGAAAACC | -2 |
| | | TCATAGACATAAGAAACCAGTCAATTACTCACAGTTTGACCACTCTGACAGTGATGGTAAGTAAAGCTTCTTATTT | WT$_{E2}$ |
| | | TCATAGACATAAGAAACCAGTCAATTACTCACAGTTTGACCACT---------------------------TATTT | -27 |
| | | | |
| *RAD54L*<br>3<br>Chr. 1 | R54L<br>KO<br>(Hs578T) | GCCTTCCAGCCTGGTGAAGAACTGGTACAATGAGGTTGGGAAATGGCTCGGAGGGAGGATCCAACCTCTGGCCATC | WT |
| | | GCCTTCC--------GAAGAACTGGTACAATGAGGTTGGGAAATGGCTCGGAGGGAGGATCCAACCTCTGGCCATC | -8 |
| | | GCCTTCC-----------------------------------------------------CCAACCTCTGGCCATC | -53 |
| | | -------------------------//--------------------------------------CTCTGGCCATC | -70 |
| *RAD51AP1*<br>3<br>Chr. 12 | R54L/<br>AP1<br>KO<br>(Hs578T) | GGAGTTAAAACAAGATAAACCAAAACCTAACTTGAACAATCTCCGGAAAGAAGAAATCCCAGTACAAGAGAAAACC | WT$_{E3}$ |
| | | GGAGTTAAAACAAGATAAACCAAAACCTAACTTGAACAAT---------------ATCCCAGTACAAGAGAAAACC | -15 |
| | | GGAGTTAAAACAAGATAAACCAAAACCTAACTTGAAC----TCCGGAAAGAAGAAATCCCAGTACAAGAGAAAACC | -4 |
| | | GGAGTTAAAACAAGATAAACCAAAACCTAACTTGAACAATC--CGGAAAGAAGAAATCCCAGTACAAGAGAAAACC | -2 |

*Estimated from: (Adey et al., 2013); (Hackett et al., 1977).
#Small lettering for insertions and bp changes.
WT: wild-type sequence.

**Supplementary Table 2.** Sensitivity Enhancement Ratios (SER) for double KO (DKO)/knockdown cells, based on IC50* values for single KO/knockdown and DKO/knockdown cells.

| **R54L/AP1 DKO – MMC (Fig. 1B)** | | |
|---|---|---|
| **Cell line** | **IC50 (µM)** | **SER** |
| HeLa | 0.3902 | - |
| AP1 KO | 0.1715 | 2.28 |
| R54L KO-1 | 0.1894 | 2.06 |
| R54L KO-2 | 0.2613 | 1.49 |
| R54L/AP1 DKO-1 | 0.0890 | 4.39§ |
| R54L/AP1 DKO-2 | 0.0945 | 4.13(§) |
| | | |
| **R54L/AP1 knockdown – MMC (Fig. 1C)** | | |
| **Cell line** | **IC50 (µM)** | **SER** |
| A549 + siRNA: Ctrl | 0.5184 | - |
| A549 + siRNA: AP1 | 0.2360 | 2.20 |
| A549 + siRNA: R54L | 0.2668 | 1.46 |
| A549 + siRNA: AP1+R54L | 0.0617 | 6.33§ |
| | | |
| **R54L/AP1 DKO – Olaparib (Fig. 1E, 1F)** | | |
| **Cell line** | **IC50 (µM)** | **SER** |
| HeLa | 1.5190 | - |
| AP1 KO | 0.9567 | 1.59 |
| R54L KO-1 | 0.6247 | 2.43 |
| R54L KO-2 | 0.5191 | 2.93 |
| R54L/AP1 DKO-1 | 0.1373 | 11.06§ |
| R54L/AP1 DKO-2 | 0.1160 | 13.09§ |
| | | |
| Hs578T | 1.690 | - |
| AP1 KO | 1.1110 | 1.52 |
| R54L KO | 0.6403 | 2.64 |
| R54L/AP1 DKO | 0.06087 | 27.76§ |

§Indicates synergistic response for combined loss of function.
*Best-fit values determined by GraphPad Prism 9.

**Supplementary Table 3.** Summary of DNA fiber data analyses. Means, medians, 25th and 75th percentiles, *p*-values and number of experiments (N), as shown in the graphs of the corresponding figures.

| Fig. | Label | Genotype | Treat-ment | Mean (µm) | Median (µm) | 25th percentile | 75th percentile | *p* | N |
|---|---|---|---|---|---|---|---|---|---|
| **3B** | IdU | WT | HU | 5.94 | 5.67 | 4.74 | 6.82 | - | 3 |
| **3B** | IdU | AP1 KO | HU | 5.81 | 5.50 | 4.43 | 6.77 | >0.9999§ | 3 |
| **3B** | IdU | R54L KO-1 | HU | 7.26 | 6.77 | 5.63 | 8.63 | <0.0001§ | 3 |
| **3B** | IdU | R54L/AP1 DKO-1 | HU | 3.23 | 3.07 | 2.32 | 3.93 | <0.0001§,† | 3 |
| **3C** | IdU | WT | NT | 10.05 | 9.86 | 8.97 | 10.84 | - | 3 |
| **3C** | IdU | AP1 KO | NT | 10.37 | 10.14 | 9.25 | 11.30 | 0.0394§ | 3 |
| **3C** | IdU | R54L KO-1 | NT | 10.00 | 10.04 | 9.01 | 11.05 | >0.9999§ | 3 |
| **3C** | IdU | R54L/AP1 DKO-1 | NT | 9.53 | 9.34 | 8.46 | 10.45 | <0.0001§ <0.0001† | 3 |
| **3D** | CldU | WT | HU | 7.39 | 7.19 | 6.14 | 8.47 | - | 3 |
| **3D** | CldU | AP1 KO | HU | 8.14 | 7.99 | 6.96 | 9.15 | <0.0001§ 0.0061† | 3 |
| **3D** | CldU | R54L KO-1 | HU | 8.54 | 8.42 | 7.42 | 9.61 | <0.0001§ | 3 |
| **3D** | CldU | R54L/AP1 DKO-1 | HU | 8.22 | 8.09 | 6.86 | 9.42 | <0.0001§ <0.0143† | 3 |
| **3E** | CldU | WT | NT | 9.88 | 9.91 | 8.94 | 10.84 | - | 3 |
| **3E** | CldU | AP1 KO | NT | 10.57 | 10.00 | 8.65 | 11.66 | >0.5523§ 0.1721† | 3 |
| **3E** | CldU | R54L KO-1 | NT | 10.30 | 10.23 | 9.31 | 11.23 | 0.0017§ | 3 |
| **3E** | CldU | R54L/AP1 DKO-1 | NT | 9.70 | 9.62 | 8.75 | 10.53 | >0.1929§ <0.0001† | 3 |
| **4F** | IdU | WT | NT | 10.15 | 10.03 | 9.09 | 11.10 | - | 3 |
| **4F** | IdU | R54L/AP1 KO-1 | NT | 9.34 | 9.17 | 8.27 | 10.16 | <0.0001§,¶ | 3 |
| **4F** | IdU | R54L/R54B DKO-1 | NT | 9.73 | 9.73 | 8.92 | 10.53 | <0.0001§ | 3 |
| **4F** | IdU | R54L/R54B DKO-2 | NT | 10.53 | 10.46 | 9.69 | 11.11 | | 1 |
| **4G** | IdU | WT | HU | 5.44 | 5.22 | 4.63 | 6.13 | - | 3 |

| | | | | | | | | | |
|---|---|---|---|---|---|---|---|---|---|
| **4G** | IdU | R54L/AP1 KO-1 | HU | 3.12 | 3.03 | 2.33 | 3.80 | <0.0001[§,¶] | 3 |
| **4G** | IdU | R54L/R54B DKO-1 | HU | 4.79 | 4.56 | 3.87 | 5.51 | <0.0001[§] | 3 |
| **4G** | IdU | R54L/R54B DKO-2 | HU | 4.83 | 4.46 | 3.64 | 5.69 | | 1 |
| **5E** | CldU+ IdU | WT | Olaparib | 16.63 | 15.85 | 12.83 | 19.56 | - | 3 |
| **5E** | CldU+ IdU | AP1 KO | Olaparib | 19.72 | 19.55 | 16.79 | 22.20 | <0.0001[§,†] | 3 |
| **5E** | CldU+ IdU | R54L KO-1 | Olaparib | 23.57 | 23.90 | 20.97 | 26.71 | <0.0001[§,$] | 3 |
| **5E** | CldU+ IdU | R54L/AP1 DKO-2 | Olaparib | 22.04 | 22.01 | 18.93 | 25.22 | <0.0001[§,†,$] 0.1822[¶] | 3 |
| **5E** | CldU+ IdU | R54L/R54B DKO-1 | Olaparib | 21.03 | 21.11 | 17.97 | 24.16 | <0.0001[§,†] 0.0091[$] | 3 |
| **5F** | CldU+ IdU | WT | NT | 13.82 | 13.69 | 12.31 | 15.13 | - | 3 |
| **5F** | CldU+ IdU | AP1 KO | NT | 13.94 | 13.48 | 11.90 | 15.51 | >0.9999[§,†] | 3 |
| **5F** | CldU+ IdU | R54L KO-1 | NT | 18.72 | 18.54 | 16.08 | 21.38 | <0.0001[§] | 3 |
| **5F** | CldU+ IdU | R54L/AP1 DKO-2 | NT | 13.02 | 13.02 | 11.52 | 14.43 | <0.0001[§,†] 0.0004[$] <0.0001[¶] | 3 |
| **5F** | CldU+ IdU | R54L/R54B DKO-1 | NT | 15.14 | 14.70 | 12.06 | 17.73 | <0.0001[§,†,$] | 3 |
| **S4G** | CldU | WT | NT | 9.90 | 9.91 | 8.94 | 10.83 | - | 3 |
| **S4G** | CldU | R54L/AP1 DKO-1 | NT | 9.62 | 9.52 | 8.69 | 10.45 | 0.0045[§] 0.0008[¶] | 3 |
| **S4G** | CldU | R54L/R54B DKO-1 | NT | 9.20 | 9.20 | 8.02 | 10.10 | <0.0001[§] | 3 |
| **S4G** | CldU | R54L/R54B DKO-2 | NT | 9.07 | 9.10 | 8.32 | 9.90 | | 1 |
| **S4H** | CldU | WT | HU | 7.46 | 7.29 | 6.33 | 8.47 | - | 3 |
| **S4H** | CldU | R54L/AP1 DKO-1 | HU | 8.25 | 8.09 | 6.86 | 9.44 | <0.0001[§,¶] | 3 |
| **S4H** | CldU | R54L/R54B DKO-1 | HU | 9.24 | 9.15 | 7.77 | 10.67 | <0.0001[§] | 3 |
| **S4H** | CldU | R54L/R54B DKO-1 | HU | 9.23 | 9.15 | 7.77 | 10.67 | | 1 |

[§] Compared to wild type (WT) HeLa cells.
[$] Compared to AP1 KO cells.
[†] Compared to RAD54L KO-1 cells.
[¶] Compared to RAD54L/RAD54B DKO-1 cells.

**Supplementary Table 4.** Summary of comet assay analyses. Means, medians, 25th and 75th percentiles, *p*-values and number of experiments (N), as shown in the graphs of the corresponding figures.

| Fig. | Genotype | Treatment | Mean (µm) | Median (µm) | 25th percentile | 75th percentile | *p* | N |
|---|---|---|---|---|---|---|---|---|
| **S5G** | WT | NT | 59.78 | 56.76 | 44.68 | 73.43 | - | 3 |
| **S5G** | AP1 KO | NT | 70.22 | 70.28 | 54.19 | 86.55 | <0.0001§ | 3 |
| **S5G** | R54L KO-1 | NT | 69.68 | 73.05 | 54.11 | 84.60 | <0.0001§<br>>0.9999$ | 3 |
| **S5G** | R54L/AP1 KO-2 | NT | 66.82 | 67.73 | 46.96 | 83.54 | 0.0002§<br>0.3579$<br>0.2999†<br>>0.9999¶ | 2 |
| **S5G** | R54L/R54B KO-1 | NT | 64.43 | 60.01 | 46.40 | 80.08 | 0.1194§<br>0.0014$<br>0.0010† | 2 |
| **S5H** | WT | Olaparib | 74.76 | 76.77 | 60.65 | 90.64 | - | 3 |
| **S5H** | AP1 KO | Olaparib | 82.92 | 83.40 | 70.08 | 95.90 | 0.0002§ | 3 |
| **S5H** | R54L KO-1 | Olaparib | 77.73 | 80.63 | 59.91 | 93.62 | 0.2355§<br>0.1325$ | 3 |
| **S5H** | R54L/AP1 KO-2 | Olaparib | 97.12 | 97.52 | 86.22 | 110.19 | <0.0001§,$,†<br>0.5380¶ | 2 |
| **S5H** | R54L/R54B KO-1 | Olaparib | 92.96 | 95.78 | 83.23 | 106.72 | <0.0001§,$,† | 2 |

§ Compared to wild type (WT) HeLa cells.
$ Compared to AP1 KO cells.
† Compared to RAD54L KO-1 cells.
¶ Compared to RAD54L/RAD54B KO-1 cells.

**Supplementary Table 5.** sgRNAs used in this study.

| Name | Target | Sequence (listed 5´- 3´) |
|---|---|---|
| sgRNA A | *RAD54L* (exon 8) | CGGAGGGAGGATCCAACCTC |
| sgRNA B | *RAD54L* (exon 8) | GTACCAGTTCTTCACCAGGC |
| sgRNA A | *RAD54B* (exon 6) | TAAGAACTGTTTCCCTCTTG |
| sgRNA B | *RAD54B* (exon 6) | TGGTGATTCTTATCTGGTCG |
| sgRNA (1) | *RAD51AP1* (exon 2) | TTTGACCACTCTGACAGTGA |
| sgRNA (2) | *RAD51AP1* (exon 3) | AACCTAACTTGAACAATCTC |
| sgRNA (3) | *RAD51AP1* (exon 5/6) | AGTGTAGCCAGTGATTATTT |

**Supplementary Table 6.** PCR primers used in this study.

| Primer | Target | Sequence (listed 5´- 3´) | Product Length (bp) |
|---|---|---|---|
| P1 | *RAD54L* (intron 7) | AGACTACCATCCCTGGGACA | 597 |
| P2 | *RAD54L* (intron 8) | CAACAGAAAAGGTGTAAAGGGAACA | |
| P3 | *RAD54L* (exon 7) | GTTGACCCTATTCTCAGTAAGGTT | 883 |
| P4 | *RAD54L* (exon 9) | CCACGCTGGTTCATGAATCCTT | |
| P5 | *RAD54B* (intron 5) | TGAGAAGCTGTGAACATTGGC | 894 |
| P6 | *RAD54B* (intron 6) | CCACACTAGCAGTCGGTAAG | |
| P7 | *RAD54B* (intron 5) | CGTTCATCAGTAAGGCATGAGA | 598 |
| P8 | *RAD54B* (intron 6) | CCGTGGTCCTGATTCCCATA | |
| P9 | *RAD51AP1* (intron 1) | TCCCCGCGGTAAAATGCAAATC | 496 |
| P10 | *RAD51AP1* (intron 2) | CACCTGGCCTGTTCATTTATCACC | |
| P11 | *RAD51AP1* (intron 2) | AGGGCACAAAAACAAAAGTCGA | 470 |
| P12 | *RAD51AP1* (intron 3) | CGTCCTGTTTTCTGACTGCACC | |
| P13 | *RAD51AP1* (intron4/5) | TGCCAGTTGGAGTTTGGGATCA | 420 |
| P14 | *RAD51AP1* (intron5/6) | AAGCCACGGGTAGTTATGACCC | |

**Supplementary Table 7.** siRNAs used in this study.

| Name | Target Sequence (listed 5´- 3´) |
|---|---|
| non-depleting control (Parplys et al., 2015) | GATTCGAACGTGTCACGTCAA |
| *RAD51AP1* (Parplys et al., 2015) | AACCTCATATCTCTAATTGCA |
| *RAD54L* (Maranon et al., 2020) | AAGCATTTATTCGAAGCATTT |
| *RAD54B* | ACCCAAGAAATTATAAATAAA |
